# Supplementary material for: Protocol for the cultural adaptation of pulmonary rehabilitation and subsequent testing in a randomised controlled feasibility trial for adults with chronic obstructive pulmonary disease in Sri Lanka
Source: BMJ Open. 2020 Nov 26;10(11):e041677. doi: 10.1136/bmjopen-2020-041677 (PMC7692826; doi:10.1136/bmjopen-2020-041677)
Supplement: Supplementary data [file bmjopen-2020-041677supp001.pdf]

Appendices

Information sheet

Cultural adaptation of Pulmonary Rehabilitation and subsequent testing in a Randomised Controlled Feasibility Trial for Adults with Chronic Obstructive Pulmonary Disease in Sri Lanka

I am Prof. S, W. Wimalasekera, of Department of Physiology, Faculty of Medical Sciences, University of Sri Jayewardenepura. I and my team of health care personnel are, conducting a research on "Testing the Feasibility and Acceptability of a Culturally Appropriate Hospital-Based Pulmonary Rehabilitation (PR) Programme for Adults with Chronic Obstructive Pulmonary Disease in Sri Lanka". I invite you to participate in the study. Please take time and read the following information carefully before giving your consent. You are free to ask questions to clarify facts which are not clear or ask for more information if you need.

Purpose of the study

The purpose of this study is to devise an appropriate PR programme and then determine the feasibility and acceptability of this programme for adults living with COPD in Sri Lanka and assess the potential for a future trial of its effectiveness.

When you participate you will be required to do the following

You will be requested to participate several components of the study.

You will participate in a group discussion conducted in a quiet room in the clinic (conference hall) with very minimal disturbances. At the beginning of the discussion you will answer some questions regards your personal information, (age, gender, history of the disease condition). Then the interviewer will ask some questions regarding your thoughts and ideas about pulmonary rehabilitation. You and your group members can freely comment on the questions or can add your ideas to the discussion. The interview takes around forty-five minutes of time.

You will also participate in a feasibility trial which you will be allocated to one of the two groups. One group will receive usual care and PR and the other group will receive only usual care which consists of pharmacological treatment and brief information about disease condition, medication and inhaler techniques. PR is typically a 6-week rolling programme and consisting of 12 sessions in total, delivered twice a week. Sessions usually last approximately two hours, with one hour for exercise training and one hour for education. Some routine and specific test and measurements will be taken before and after the intervention will describe any changes in the health of the adults living with COPD.

If you are allocated to the group who receive the PR, you will be asked to share your experiences regarding the PR and subjected modification and opinions on pulmonary rehabilitation.

In order to maintain accuracy, and to keep records, your discussion will be recorded by the research team members with your permission. Your participation in the project will remain confidential and the provision of your name and address is necessary for us to maintain records. Any personal information gathered during the study will not be disclosed to a third party. Your responses to the questions and will

only be used for the purpose of this project. You can be assured that you will remain anonymous if you take part in the project. Your participation in this project is entirely voluntary. You are not obliged to take part. If you do not wish to take part, you can withdraw without giving a reason and your medical care will not be affected, further you will not be contacted again. Similarly, if you do agree to participate, you are free to withdraw at any time during the project.

What are the possible benefits of taking part?

By participating in the study, you will contribute to develop a PR programme which helps to improve the quality of life of the adults living with COPD. As well as you will contribute to determine the feasibility and acceptability of this programme within the health care system in Sri Lanka.

What are the possible disadvantages and risks of taking part?

You will be undertaking some physical tests as part of the research. Therefore, there may be a very small risk of falls, breathlessness, changes in blood pressure and changes in heart rate. However, these risks are very rare, and trained staff and emergency equipment will be available to deal with any serious events. Participants who experience any such event will be directed to the appropriate hospital and all the necessary care will be ensured and followed-up until the participant has resolved or stabilized.

What will happen if you don't carry on with the study?

If you withdraw from the study all the information and data collected from you, to date, will be destroyed and your name would be removed from all the research study files. There will not be any loss or impact on your routine medical care which you are entitled to.

What will happen to the results of the research study?

The results of all the participants of the study will be used for scientific research. All data will be available only to the researchers and information provided will be strictly confidential. Your participation in the study is highly appreciated. Privacy and confidentiality of data gathered is completely secure and will be used only for research purposes. If you have any questions or any concerns with regards to the study and need further information you can call the telephone number listed below. Further you can send any complaint to the above postal address or email address.

Yours faithfully

Prof. S.W. Wimalasekera

Department of Physiology, Faculty of Medical Sciences, University of Sri Jayewardenepura

T.P No. 0777487203 e-mail : savithriww@yahoo.com

Ethics Review Committee – University of Sri Jayewardenepura - +94 11 2758000 Ext 4075e

Consent Form: **Randomised Controlled Feasibility Trial**

Cultural adaptation of Pulmonary Rehabilitation and subsequent testing in a Randomised Controlled Feasibility Trial for Adults with Chronic Obstructive Pulmonary Disease in Sri Lanka

1.Have you read the information sheet? (please keep a copy for yourself) YES/NO

2.Have you had an opportunity to discuss this and ask any questions? YES/NO

3. Have you had satisfactory answers to all your questions? YES/NO

4. Have you received enough information about the study? YES/NO

5. Who explained the study to you--

6. Do you understand that you are free to withdraw from the study at any time,

without having to give a reason YES/NO

7. All personal details will be treated as STRICTLY CONFIDENTIAL.

Do you give your permission for these individuals to have access to the records? YES/NO

8. Have you had sufficient time to come to your decision? YES/NO

9. Do you agree to take part in this study? YES/NO

Participant's signature --------------------------------------- Date------------------------

By the Witness

I have been present while the procedure was explained to the participant and I have witness his/ her willingness to take part in the study.

Signature of witness--- Date-------------------------------

(The witness should be a person not connected to the study)

Name --

Contact details---
