# Supplementary material for: Protocol for the cultural adaptation of pulmonary rehabilitation and subsequent testing in a randomised controlled feasibility trial for adults with chronic obstructive pulmonary disease in Sri Lanka
Source: BMJ Open. 2020 Nov 26;10(11):e041677. doi: 10.1136/bmjopen-2020-041677 (PMC7692826; doi:10.1136/bmjopen-2020-041677)
Supplement: Author's manuscript [file bmjopen-2020-041677.draft_revisions.pdf]

BMJ Open

BMJ Open is committed to open peer review. As part of this commitment we make the peer review history of every article we publish publicly available.

When an article is published we post the peer reviewers' comments and the authors' responses online. We also post the versions of the paper that were used during peer review. These are the versions that the peer review comments apply to.

The versions of the paper that follow are the versions that were submitted during the peer review process. They are not the versions of record or the final published versions. They should not be cited or distributed as the published version of this manuscript.

BMJ Open is an open access journal and the full, final, typeset and author-corrected version of record of the manuscript is available on our site with no access controls, subscription charges or pay-per-view fees (http://bmjopen.bmj.com).

If you have any questions on BMJ Open's open peer review process please email info.bmjopen@bmj.com

Protocol for the cultural adaptation of Pulmonary Rehabilitation and subsequent testing in a Randomised Controlled Feasibility Trial for Adults with Chronic Obstructive Pulmonary Disease in Sri Lanka

Journal:	*BMJ Open*
Manuscript ID	bmjopen-2020-041677
Article Type:	Protocol
Date Submitted by the Author:	14-Jun-2020
Complete List of Authors:	Jayamaha, Akila; KIU, Health Sciences<br>Perera, Chamilya; KIU, Health Sciences<br>Orme, Mark; University Hospitals of Leicester NHS Trust, Centre for Exercise and Rehabilitation Science, NIHR Leicester Biomedical Research Centre-Respiratory; University of Leicester, Department of Respiratory Sciences<br>Jones , Amy; University Hospitals of Leicester NHS Trust, Centre for Exercise and Rehabilitation Science, NIHR Leicester Biomedical Research Centre-Respiratory; University of Leicester, Department of Respiratory Sciences<br>Wijayasiri, Upendra; Colombo South Teaching Hospital, Sports Medicine Unit<br>Amarasekara, Thamara; University of Sri Jayewardenepura, Faculty of Allied Health Sciences<br>Karunatillake, Ravini ; National Hospital of Sri Lanka, Central Chest Clinic<br>Fernando, Amitha; National Hospital of Sri Lanka, Central Chest Clinic<br>Seneviratne, Anthony ; Primary Care Respiratory Group<br>Barton, Andy; University of Plymouth, Faculty of Health<br>Jones, Rupert; University of Plymouth, Faculty of Health<br>Miah, Ruhme ; University of Leicester, Department of Respiratory Sciences; University Hospitals of Leicester NHS Trust, Centre for Exercise and Rehabilitation Science, NIHR Leicester Biomedical Research Centre-Respiratory<br>Malcolm, Dominic; Loughborough University, School of Sport, Exercise and Health Sciences<br>Matheson, Jesse; University of Sheffield, Department of Economics<br>Free, Robert; University of Leicester, Department of Respiratory Sciences,<br>Manise, Adrian ; University of Leicester, Department of Respiratory Sciences<br>Steiner, michael ; University of Leicester, Department of Respiratory Sciences; University Hospitals of Leicester NHS Trust, Centre for Exercise and Rehabilitation Science, NIHR Leicester Biomedical Research Centre-Respiratory<br>Wimalasekera, Savithri; University of Sri Jayewardenepura, Faculty of Medical Sciences<br>Singh, Sally; University of Leicester, Department of Respiratory Sciences; University Hospitals of Leicester NHS Trust, Centre for Exercise

	and Rehabilitation Science, NIHR Leicester Biomedical Research Centre-Respiratory
Keywords:	REHABILITATION MEDICINE, Chronic airways disease < THORACIC MEDICINE, Emphysema < THORACIC MEDICINE

SCHOLARONE™ Manuscripts

I, the Submitting Author has the right to grant and does grant on behalf of all authors of the Work (as defined in the below author licence), an exclusive licence and/or a non-exclusive licence for contributions from authors who are: i) UK Crown employees; ii) where BMJ has agreed a CC-BY licence shall apply, and/or iii) in accordance with the terms applicable for US Federal Government officers or employees acting as part of their official duties; on a worldwide, perpetual, irrevocable, royalty-free basis to BMJ Publishing Group Ltd ("BMJ") its licensees and where the relevant Journal is co-owned by BMJ to the co-owners of the Journal, to publish the Work in this journal and any other BMJ products and to exploit all rights, as set out in our licence.

The Submitting Author accepts and understands that any supply made under these terms is made by BMJ to the Submitting Author unless you are acting as an employee on behalf of your employer or a postgraduate student of an affiliated institution which is paying any applicable article publishing charge ("APC") for Open Access articles. Where the Submitting Author wishes to make the Work available on an Open Access basis (and intends to pay the relevant APC), the terms of reuse of such Open Access shall be governed by a Creative Commons licence – details of these licences and which Creative Commons licence will apply to this Work are set out in our licence referred to above.

Other than as permitted in any relevant BMJ Author's Self Archiving Policies, I confirm this Work has not been accepted for publication elsewhere, is not being considered for publication elsewhere and does not duplicate material already published. I confirm all authors consent to publication of this Work and authorise the granting of this licence.

For peer review only

Protocol for the cultural adaptation of Pulmonary Rehabilitation and subsequent testing in a Randomised Controlled Feasibility Trial for Adults with Chronic Obstructive Pulmonary Disease in Sri Lanka

Akila R Jayamaha [1], Chamilya H Perera [1], Mark W Orme [2,3], Amy V Jones [2,3], Upendra K D C Wijayasiri[4], Thamara D Amarasekara[5], Ravini de S Karunatillake [6], Amitha C Fernando [6], Seneviratne A L P De S.[7], Andy Barton [8], Rupert Jones [8], Ruhme B Miah[2,3], Dominic Malcolm[9], Jesse A Matheson [10], Robert C Free[3], Adrian Manise[3], Michael C Steiner[2,3] Savithri W Wimalasekera [11], Sally J Singh [2,3]

Correspondence to Akila Randika Jayamaha/1226arj@gmail.com/akila@kiu.ac.lk

For peer review only

Abstract

Introduction International guidelines recommend pulmonary rehabilitation (PR) should be offered to adults living with COPD, but there is limited PR available in Sri Lanka. Culturally appropriate PR needs to be designed and implemented in Sri Lanka. The study aims to adapt PR to the Sri Lankan context and determine the feasibility of conducting a future trial of the adapted PR in Sri Lanka.

Methods and analysis Phase-1: Develop the intervention: Focus groups with adults living with COPD and caregivers and in-depth interviews with healthcare professionals will be conducted to inform PR adaptations. Phase-2: Randomised controlled feasibility trial conducted in Central Chest Clinic, Colombo, Sri Lanka. Eligible participants will be identified and will be invited to take part. A total of 60 participants will be recruited to the trial and randomised (1:1) into one of two groups; control group receiving usual care or the intervention group also receiving PR. The PR programme is likely to consist of 12 sessions of exercises and health education, delivered twice weekly. After completion of PR, routine measures in both groups will be assessed by a blinded assessor. The primary outcome measure is feasibility, including assessing eligibility, uptake and completion. Phase-3: Qualitative evaluation of the trail: Focus groups with participants and in-depth interviews with PR deliverers will be conducted to further determine feasibility and acceptability of PR, as well as ability to run a larger trial.

Ethics and dissemination Ethical approval will be obtained from the ethics review committee of the Faculty of Medical Sciences, University of Sri Jayewardenepura, Sri Lanka and the University of Leicester, UK. The results of the trial will be disseminated through patient and

For peer review only - http://bmjopen.bmj.com/site/about/guidelines.xhtml

public involvement events, local and international conference proceedings, and peer-reviewed journals.

Trial registration: Phase-1) ISRCTN58273367, Phases-2 &3) ISRCTN13367735

Keywords: COPD, Pulmonary Rehabilitation, Exercise, Education, Sri Lanka

Strengths and limitations

- This study is the first examining the feasibility and acceptability of a culturally appropriate pulmonary rehabilitation programme for adults with COPD in Sri Lanka.
- Taking a mixed-method approach, this study will provide a rich insight into delivering a trial of pulmonary rehabilitation in the Sri Lankan context.
- This study is a single center feasibility trial. As such, whilst findings will be an important first step in understanding the potential role of pulmonary rehabilitation in Sri Lanka and low- and middle-income countries more broadly, findings may not be generalizable to other regions of the world.

For peer review only

INTRODUCTION

Chronic obstructive pulmonary disease (COPD) is a progressive disease characterised by airflow obstruction and breathlessness. COPD is a major cause of morbidity and mortality throughout the world, corresponding to 6% of all deaths worldwide.[1] Further, more than 90% of COPD deaths occur in low and middle-income countries.[2] The most recent estimate of COPD prevalence in South Asia was 6.3%[3] with a prevalence in Sri Lanka of 10.5%,[4] similar to the estimated global COPD prevalence of 11.7%.[5] COPD is a significant burden to both patients and healthcare services.[6]

Chronic cough with sputum, breathlessness, physical inactivity and exercise intolerance resulting from dyspnoea or fatigue are common consequences of COPD.[1] Symptoms of COPD progressively worsen and people can become breathless, even at rest. Daily activities often become difficult as the condition worsens, impacting their quality of life.[7] The impact of COPD to the individual and to society makes the need for interventions to reverse the associated disability of paramount importance.

The development of rehabilitation in low-income and middle-income countries will ensure the Universal Health Coverage.[8] International guidelines recommend that pulmonary rehabilitation (PR) should be routinely offered to patients with chronic respiratory disease who have persistent symptoms, limited activity, and/or are unable to adjust to illness.[9] It is a low cost, high impact intervention that improves the quality of life, reduces suffering, reduces mortality and reduces economic loss, relieves dyspnoea and fatigue, improves exercise capacity, improves psychological and emotional function, and enhances an individual's self-management of their condition.[1] Having realised the benefits of PR in COPD, Western countries have incorporated this as an important structural component of healthcare delivery services.[10] Implementation of PR based as practiced in Western countries in Sri Lanka requires adaptation to the local health service, population and culture. Despite its effectiveness, there is a significant need to understand the feasibility of conducting PR in low resource Sri Lankan health care setting and the acceptability of PR among Sri Lankan adults living with COPD and healthcare staff involved in its delivery. Uptake and completion of PR even in Western countries is an on-going challenge.[11] The need to maximize appeal of PR to patients and referrers is a global issue and one that is

For peer review only - http://bmjopen.bmj.com/site/about/guidelines.xhtml

likely to be specific to a given location and population. There remains an unmet need for PR in Sri Lanka. To be successful, PR must be not only evidence-based, but also designed and implemented in a manner sensitive to the context in which it is being delivered, such as culture and geography. Therefore, the aim of this study is to devise an appropriate PR programme and then determine the feasibility and acceptability of this programme for adults living with COPD in Sri Lanka and assess the potential for a future trial of its effectiveness.

The objectives of the study are to:

1. Explore the needs and perceptions of adults living with COPD, their care-givers /family members and health care professionals to inform the adaptations required for a PR programme suitable for the Sri Lankan context.
2. Determine the feasibility of conducting adapted hospital-based PR for people living with COPD.
3. Assess the acceptability of the PR among Sri Lankan adults living with COPD and healthcare staff involved in its delivery.
4. Describe any changes in health of the adults living with COPD following completion of PR.
5. Assess the feasibility of a future trial and estimate the required sample size.

METHODS AND ANALYSIS

Study design and registration

The proposed study will be conducted in three phases. Phase 1: A qualitative study will be conducted to inform the adaptations required to make PR specific to the Sri Lankan context. Phase 2 is a single blind randomised control feasibility trial. Adults living with COPD will be randomised (1:1) into adapted PR or usual care. Phase 3: Qualitative evaluation of the trial to determine the feasibility and acceptability of PR deliverers and participants. The trial will be conducted, analysed and reported according to the Standard Protocol Items: Recommendations for Interventional Trials (SPIRIT) statement[12] and trial has been registered on the ISRCTN website. The design of the study and flow of participant enrolment is presented in figure 1.

For peer review only - http://bmjopen.bmj.com/site/about/guidelines.xhtml

Study setting

The study will be carried out at the Central Chest Clinic, Colombo, Sri Lanka which is a leading health care facility that provides treatment for adults with respiratory disease. A room allowing confidential discussions will be used for conducting focus group discussions among adults living with COPD, their caregivers /family members, and nurses. Semi structured interviews will be conducted among doctors and physiotherapists in the conference hall (which is quiet and not used routinely for work) or consultation rooms as convenient to them and without interfering with their routine work. The venue for PR will be a large room at the Central Chest Clinic.

Participants

Key informants and suitable participants for phase 1 of the study will be identified by the researchers with the help of health care professionals involved in the treatment of COPD at Central Chest Clinic. Suitable participants (adults living with COPD, their caregivers /family members, nurses, doctors, physiotherapists) will be selected using purposive sampling method. Multi-religious and multi-ethnic Sri Lankan cultural diversity and gender will be considered when recruiting to the study. Participants who provide written informed consent will be enrolled in the study.

After the adaptation of PR in phase 1, suitable participants for the Phase 2 randomised controlled feasibility trial will be identified by the Medical Officers in the Chest Clinic and will be invited to take part in an eligibility assessment. This assessment will determine eligibility for entry into the study. People eligible for inclusion in the trial will be: aged ≥18 years, will have a clinically confirmed diagnosis of COPD by a physician, confirmed COPD with spirometry based on GOLD criteria with $FEV_1/FVC<0.7$, and $FEV_1<80\%$ predicted, ≥1 exacerbation per year, mMRC grade ≥2 and willing to provide informed consent. Adults with comorbidities such as severe or unstable cardiovascular, other internal diseases and locomotor difficulties that preclude the exercise or malignant disease or other serious illness which will interfere with participation in the PR, will be excluded from the study. Individuals not eligible for the study will be recorded on a study screening log.

For peer review only - http://bmjopen.bmj.com/site/about/guidelines.xhtml

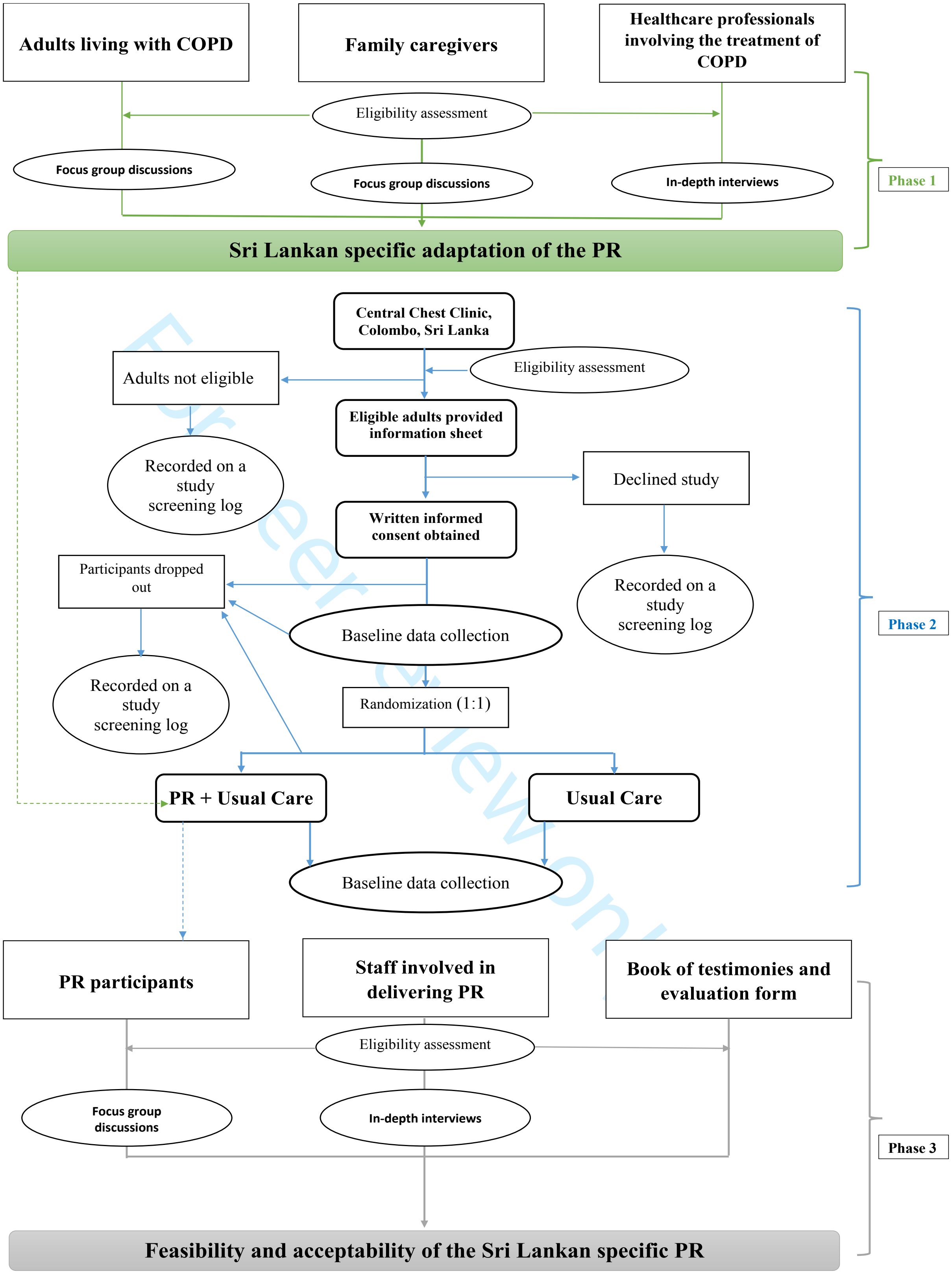

Figure 1: Flow of participant of the three phases of the study

Procedure

Eligible participants will be informed verbally about the study by the Medical Officers of the Central Chest Clinic. After receiving a study information sheet, potential participants will be contacted to arrange an appointment, if they wish to take part. An opportunity to ask questions will be provided. If willing to take part in the study, they will be asked to provide written informed consent. Information regarding the interest of participation in the study will be taken as field notes. Baseline outcome measures will be assessed. After baseline data collection, participants will be randomised (1:1) into two groups. One group will receive usual care and PR and the other group will receive only usual care which consists of pharmacological treatment and brief information about disease condition, medication and inhaler techniques. Experiences of the participants and PR deliverers regarding the acceptability and feasibility of PR will be explored in interviews and focus groups during Phase 3 of the study. Participants who did not complete the PR will be asked to take part in a drop-out interview and information provided freely by the participants will be collected as field notes.

Trial interventions

Intervention: Adapted pulmonary rehabilitation

Sri-Lankan specific PR will comprise the core elements of an evidence based rehabilitation, a programme of exercises[13, 14] and health education.[13, 14, 15] The detail of delivery and adaptations of PR will be informed by collecting the views and opinions from adults living with COPD, their family caregivers, and healthcare professionals. PR is typically a 6-week rolling programme and consisting of 12 sessions in total, delivered twice a week.[13, 14] Sessions usually last approximately two hours, with one hour for exercise training and one hour for education. The duration of the PR trial and frequency and duration of sessions will be discussed during Phase 1. All staff delivering PR will be trained and assisted by a nursing officer, a clinical nutritionist, an exercise physician and a physiotherapist. The venue for PR will be at the Central Chest Clinic, Colombo, Sri Lanka. The Central Chest Clinic will have a maximum capacity of 10 patients per PR class. The equipment required will be simple and include chairs, weights, and simple exercise equipment based on local availability, suitability and informed by qualitative work. [16]

For peer review only - http://bmjopen.bmj.com/site/about/guidelines.xhtml

In keeping with evidence-based guidelines[13, 14], discussions in Phase 1 will include the core exercise component of PR, notably resistance training (e.g. upper and lower limbs) and aerobic training (walking, sit-to-stand, step-ups) using minimal equipment and education. Each participant's exercise regimen will be individually prescribed and progressed[17] with walking speed will be prescribed using the ISWT.[18] The process in which this is achieved by patients will be informed by Phase 1. The education component will be delivered by an interdisciplinary team and will be informed by qualitative findings from Phase 1 with discussions facilitated by usual education topics as described in guidelines.[13, 14]

Usual care

Usual care will consist of pharmacological treatment and optimization, prescribed to patients after consultation with Medical Officers at the Central Chest Clinic. Brief information about disease condition, medication and inhaler techniques will be provided by Medical Officers and Nurses. Pulmonary rehabilitation or any form of exercise recommendation is not part of usual care.

Outcomes

Primary outcome

The primary outcome of the trial will be the feasibility and the acceptability of the PR intervention.

Feasibility

Measures to assess feasibility are provided in table 1 and include the comprehensive assessment of the feasibility of patient recruitment and the intervention delivery.

**Table 1: Primary outcome measures- Feasibility and operational experience assessment**	
**Feasibility of patient recruitment**	**Data sources**
Feasibility of screening and recruiting participants	Interviews with the healthcare professionals, screening log
Suitability of the inclusion criteria	Interviews with the healthcare professionals, screening log

For peer review only - http://bmjopen.bmj.com/site/about/guidelines.xhtml

Number of eligible patients, number of patients screened, number of patients invited to take part, actual number of participants who consent to take part	Screening log
Number of patients who refuse, drop out and the reasons for refuse and drop out	Interviews with the patients, screening log
**Operational experience of intervention delivery**	
Service provider and multi-disciplinary teams' willingness and ability to deliver the PR	Interviews with healthcare professionals
The practicality of delivering the intervention in the proposed setting	Interviews with healthcare professionals and focus groups with participants
The time needed to collect the data<br>Baseline visit- Time taken for each measure (each individual questionnaire and physical measure)<br>Follow-up visit- Time taken for each measure (each individual questionnaire and physical measure)	Interviews with the healthcare professionals, Rehabilitation records
Data completeness and accuracy	Interviews with the healthcare professionals, Rehabilitation records, RedCap
Adherence to home exercise	Interviews with the patients and self-report exercise diary
The training and resources needed to deliver the intervention (ensuring readiness for a future much larger multi-center trial)	Interviews with the healthcare professionals and focus groups with participants, Rehabilitation records
Description of unintended events	Adverse events log, REDCap

Acceptability

The acceptability of the PR intervention among adults living with COPD and healthcare staff involved in its delivery will be assessed. Participants' experience of the PR, including any perceived benefits, challenges and changes they would make to the programme, will be explored in qualitative interviews and focus groups after their discharge assessment or withdrawal. The experience of healthcare professionals regarding the PR intervention, such as their confidence in delivering the programme, the components of PR, structure of PR, the patient adherence to the PR exercises and how their perceptions changed over the course of the trial, will be explored in qualitative interviews at the end of the trial.

Secondary outcomes

The secondary outcomes of this study are provided in table 2. Comparison of secondary outcome measures of baseline and post intervention, will describe any changes in the health of the adults living with COPD following completion of PR

For peer review only - http://bmjopen.bmj.com/site/about/guidelines.xhtml

Table 2: Secondary outcome measures		
**Outcome measures**	**Baseline**	**Post-intervention**
Socio-demographics	x	
Lung health (spirometry data, smoking status, number of COPD exacerbations in the last year)	x	
Comorbidities	x	
Treatments	x	
Nutritional status (Body Mass Index, mid upper arm circumference, skinfold thickness, self-report seven-day diet diary)	x	x
Disease burden (MRC dyspnea grade, CAT, CCQ)	x	x
Economic impact of disease (WPAI)	x	x
Quality of life (EQ-5D-5L)	x	x
Psychological wellbeing (Hospital Anxiety and Depression scale)	x	x
Physical function (5x sit-to-stand test)	x	x
Exercise capacity (ISWT, ESWT)	x	x
Accelerometer assessed physical activity (ActiGraph wGT3x-BT)	x	x

MRC - Medical Research Council, CAT- COPD Assessment Test, CCQ - Clinical COPD Questionnaire, EQ-5D-5L - EuroQol Five Dimensions Five Levels, ISWT - Incremental Shuttle Walk Test, ESWT - Endurance Shuttle Walk Tests, WPAI – Work Productivity and Activity Impairment questionnaire

Sample size and recruitment target

This study is a feasibility trial that aims to provide data for an accurate estimation of the required sample size for future trials. Therefore, a formal sample size calculation is not required. We aim to recruit and randomise 60 participants to the study (30 in each group).

Patient allocation, concealment, and blinding

Randomisation will occur through random permuted blocks to either the intervention group (PR+ usual care) or control group (usual care) in ratio 1:1 using Sealed envelope™.

The computer-generated patient allocation sequence will be monitored by an individual who is independent of the research team and will inform the research team of group allocations via telephone. Participants will be informed about their group allocation after providing informed consent and completing baseline assessments.

For peer review only - http://bmjopen.bmj.com/site/about/guidelines.xhtml

It will not be possible to blind patients to their group allocation due to the nature of PR. Research staff will be blinded to outcome measures. Participants will be advised not to reveal their group during the follow-up assessment. Any episodes of un-blinding will be documented and reported.

Data collection

Phase 1: Qualitative assessment for adaptation of PR

Focus groups with patient and family caregivers

Focus groups with adults living with COPD and separate focus groups with their family members/ caregivers will be conducted until data saturation. We anticipate conducting up to 5 focus groups with 6-8 participants in each. Data will be collected during patients' clinic visits. Focus group discussions will be audio-recorded, expected to last approximately 45-90 minutes, and will be conducted face-to-face by an interviewer and note-taker (observer). Focus groups will be transcribed verbatim, with identifiable information removed. Envisaged outcomes of the focus groups will include Sri Lankan specific adaptation of PR.

Interviews with healthcare professionals involving the treatment of COPD

Up to 15 in-depth interviews with healthcare professionals will be conducted until data saturation. Structured interviews will be conducted with healthcare professionals as convenient to them without interfering with their routine work. Interviews will be audio-recorded and will be conducted face-to-face by an interviewer. Interviews will be transcribed verbatim, with identifiable information removed. Envisaged outcomes of the in-depth interviews will be a suitable design and content of PR acceptable respiratory health care deliverers in Sri Lanka.

Phase 2: Single blind randomised control feasibility trial

Data will be collected by trained researchers, following standard operating procedures during participants' clinic visits. Baseline and post intervention assessments will be carried out by the blinded Medical Officers consulting at the study setting.

For peer review only - http://bmjopen.bmj.com/site/about/guidelines.xhtml

Phase 3: Qualitative evaluation of the PR intervention

Focus groups with patients

Participants allocated to the intervention group will be invited to participate in focus group discussions at the end of their PR programme. Focus groups will give an insight on views, experiences, opinions and recommendations which will inform future PR programmes. We anticipate conducting up to 5 focus groups until data saturation. Each focus group discussion will be conducted with 6-8 participants in each.

Focus group discussions will be audio-recorded, expected to last approximately 45-90 minutes, and will be conducted a trained moderator and a note-taker). Focus groups will be transcribed verbatim, with identifiable information removed. Consent will be obtained from participants prior to their involvement in focus groups.

Interviews with PR staff

Health care personnel involved in delivering PR will be invited to participate in in-depth interviews at the end of the study to discuss aspects of feasibility and acceptability, such as insights into barriers and facilitators to attendance, logistical barriers of running a PR programme and their views of patients' experiences of the intervention. We anticipate conducting up to 15 interviews, each expected to last approximately 15-60 minutes. Interviews will be audio-recorded and will be conducted face-to-face by a trained interviewer. Interviews will be transcribed verbatim, with identifiable information removed.

Book of testimonies and evaluation form

Participants within PR will be asked to log their experience of PR as they progress through the programme. This will be in the form of a PR log book accessible to participants before, during and after sessions, as well as a dedicated evaluation form which will be provided as supplementary material. Staff involved in PR will also receive the same evaluation form at the end of the study.

For peer review only - http://bmjopen.bmj.com/site/about/guidelines.xhtml

Sample characteristics

Basic demographics including age, sex, religion, nationality, marital status, age of leaving full-time education, education level, ethnicity, employment status, monthly income, lung health, smoking status (packs per year), biomass fuel exposure, primary respiratory diagnosis, time since diagnosis in years, secondary respiratory diagnoses, family history of lung disease, comorbidities will be recorded at baseline.

Lung function

Spirometry (post bronchodilator Forced Expiratory Volume in the first second (FEV_1), post bronchodilator Forced vital capacity (FVC), FEV_1/FVC ratio, carboxyhemoglobin test [19], hospitalisations within the last 12 months, number of COPD exacerbations within the last 12 months, treatments will be collected as the baseline data prior to the randomisation of participants.

Nutritional status

Height (Holtain stadiometer), weight (calibrated weight scale) will be measured and body mass index will be calculated. Mid upper arm circumference of the participants will be measured using a measuring tape and skinfold thickness of seven sites (triceps, mid axillary, subscapular, chest, abdominal, supra iliac and thigh measurements) of the body will be measured using a validated caliper. Triplicated measurements will be taken by same investigator and mean value will be used for the calculation of body fat mass. Fat free mass will be calculated using Durnin and Womersley formula.[20, 21] Body composition analysis will be assessed using bioelectrical impedance (SFB7 Impedi Med dual frequency instrument, Impedi Med Limited, Australia) and software version 5.2.4.0.[22] Seven-day diet diary method will be used to assess the dietary history and calorie intake.

Disease burden

Breathlessness will be measured using MRC dyspnoea grade (5-items).[23] The health-related quality of life related to mobility, self-care, usual activities, pain/discomfort and anxiety-depression will be measured using EQ-5D-5L (25-items),[24] Clinical status of the airways, functional limitations and psychosocial dysfunction will be determined using CCQ (10 -items),[25]

For peer review only - http://bmjopen.bmj.com/site/about/guidelines.xhtml

CAT (8-items) will be used to determine the severity of the COPD,[26] Modified brief pain inventory (3-items) will determine the commonly reported chest pain, WPAI (8-items) will be used to measure the effect of severity of COPD and general health on work productivity and regular activities [27] and HADS (14-items) will be used to measure the patient's emotional state and the presence or absence of clinically significant anxiety and depression.[28] Disease burden will be measured as baseline data prior to the randomisation and post-intervention data.

Exercise capacity

Following a safety assessment for absolute and relative contraindications for field walking tests[29], maximal exercise capacity will be assessed using the incremental shuttle walking test (ISWT)[30] and endurance shuttle walking test (ESWT).[18] The ISWT and ESWT also include a pre-test and post-test measure of SpO2, blood pressure, Borg rating of breathlessness [31], heart rate, Borg rate of perceived exertion.[32] The sit-to stand test will be completed for five continuous repetitions, to measure lower limb movement and strength.[33] All measures of exercise capacity will be measured at baseline prior to the randomisation and post-intervention.

Accelerometry-derived physical behaviours

Participants will be asked to wear an activity monitor (ActiGraph wGT3X-BT, ActiGraph, Pensacola, FL, USA), able to detect a range of PA intensities [34], on the right anterior hip during waking hours for seven days prior to attending PR (baseline) and for the seven consecutive days prior to their discharge assessment (follow-up); taking it off for water-based activities and sleep. The collection of physical activity data will coincide with a 7-day diet diary. Printed instructions will be provided to the participants regarding how to wear the monitor.

Full accelerometry methodology is provided in Table 3. Data will be processed as 60-s epoch files in ActiLife version 6.13.4 (ActiGraph, Pensacola, FL, USA). Non-wear will be defined as 60-minutes of consecutive zero counts with allowance for 2-minutes of interruptions.[35] Adherence to wearing the monitors will be assessed by examining the proportion of participants providing valid accelerometer data across a range of valid days (≥1-7 days) and minimum wear time (≥1-12 hours) thresholds. For reported group averages, only participants providing ≥4 valid days of ≥8 hours, for both time points will be included in the analyses.[36] Step count, time spent in different absolute intensity classifications of physical behaviours[37] and average movement

For peer review only - http://bmjopen.bmj.com/site/about/guidelines.xhtml

intensity (activity counts per minute) will be reported. Time spent in physical activity matching participants' prescribed walking exercise intensity will be derived by aligning the average walking speed during the ESWT with the activity monitor counts per minute.

**Table 3: Accelerometry data collection and processing parameters**	
Accelerometer Model	ActiGraph wGT3X-BT (version 6.13.4; firmware 1.9.2)
Serial number range	Twenty unique devices will be used ranging from MOS2E09190601 to MOS2E24190146 averaging six deployments per device (same serial used for baseline and follow-up wear periods)
Piezosensor orientation	Triaxial
Mode setup	Mode 29 (x, y, z, steps, lux)
Original sample rate	100 Hz (.gt3x file format)
Deployment method	Baseline:<br>Fitted by research team on Day 0 (Baseline PR Assessment)<br>Fitted by participant on Day 1<br>Follow-up:<br>Fitted by research team Day 0 ($11^{th}/12^{th}$ session)<br>Fitted by participant on Day 1
Location worn	Anterior hip adjacent to the mid-line of the thigh
Requested days of wear	7 days of free-living (Day 1-7; 10,080 epochs)
Initialization	Not deployed in delay mode in order to standardised capture of Day 0 (00:00) with stop time based on date of first PR class (baseline) and data of discharge assessment (follow-up)
Wear instructions	Wear continuously except for sleep and water based activities
Non-wear appropriation	≥60 min of consecutive 0s with allowance for 2 minutes of interruptions were deemed biologically implausible and coded as non-wear
Valid day criteria	≥8 hours of valid waking wear time
Valid file	≥4 valid days for each of the two time points
Missing data	Data modelling or imputation will not be performed
Epoch length	60 seconds
Intensity classification (absolute)	Uniaxial (x-axis) cut-points as follows: Sedentary time <100 cpm; Light intensity 100-2019 cpm; Moderate intensity 2020-5998cpm; Vigorous intensity ≥5999cpm (Moderate-to-vigorous intensity ≥2020 cpm)
Intensity classification (relative)	Uniaxial (x-axis) cut-points based on Endurance Shuttle Walk Test performance

For peer review only - http://bmjopen.bmj.com/site/about/guidelines.xhtml

Costing

As part of this trial, the cost of creating a Sri-Lankan adapted PR programme will be calculated. This will include both single and recurrent costs. Single payments include the necessary costs to set up and run a programme, whilst recurrent cost refers to any item with a life expectancy of 1 year or less and typically includes disposable materials.[38] The fixed costs will be captured prior to the first participant enrolling into the programme and the recurrent costs will be collected at the mid-stage of recruitment. An average cost per participant will be calculated. Table 4 demonstrates the variables that will be used to calculate fixed and recurrent costs.

Table 4: The variables used to calculate fixed and recurrent costs (not an exhaustive list)

Fixed costs	Recurrent costs
Venue hire	Venue hire
Electrical equipment (laptop, printer, projector)	Staff time to conduct PR (assessment at baseline and discharge, conduct PR classes, telephone calls and data entry)
Equipment for PR (weights, treadmill, cycle ergometer, country-specific equipment, step-up box, chairs)	Disposable equipment (for blood glucose monitor, spirometer mouthpieces, nose-clips, glyceryl trinitrate spray)
Equipment for shuttle walking tests (cones, licences, stop watches, tape measure, electrical equipment to play audio)	Servicing costs (spirometer, PR equipment, specifically treadmills and cycle ergometers)
Equipment for PR assessment (height stadiometer, weight scales, sphygmomanometer, pulse oximeter, spirometer, calibration syringe, country-specific equipment)	Miscellaneous (Oxygen cylinders, questionnaire licences, stationery (paper))
Additional safety equipment (blood glucose monitor, Oxygen cylinder holder)	
Miscellaneous (filing cabinets, storage units, questionnaire translations, questionnaire licences, staff uniform)	

For peer review only - http://bmjopen.bmj.com/site/about/guidelines.xhtml

Data management

Data collected during the study will be entered into a database using Research Electronic Data Capture (REDCap), which is a web-based platform.[39] Access to the database will be via a secure password protected web-interface. The participants will be identified by a study-specific identification code. Data will be validated using real-time data entry validation and electronic checks lead by the Independent Data Monitoring Committee (IDMC), established at the University of Leicester, UK.

Quantitative data analysis

The data will be analyzed using IBM SPSS Statistics for Windows, Version 23.0. Data for baseline and follow-up time-points will be presented as descriptive statistics as appropriate. No inferential statistics will be performed due to the feasibility design of the trial.

Qualitative data analysis

Qualitative data will be analyzed using Thematic Analysis. This approach follows six distinct stages: familiarization with data; generating initial codes; searching for themes; reviewing themes; defining and naming themes and producing the report.[40] The responsible investigator will carry out initial coding and a sample of focus group transcripts will be coded by a second member of the team to improve consistency and to enhance interpretive authenticity. Throughout the data analysis, the team will meet to discuss and review emerging themes and search the accounts that provide contesting views of the same phenomena. Close attention will be paid to the complexity and interactions inherent in the focus group data.[41]

Adverse events

All adverse events and serious adverse events will be recorded on an adverse event log, within study trial management paperwork, case report forms and REDCap. There will be no formal interim analysis of data due to the feasibility nature of the trial. The IDMC will review high level safety data. Adverse events will be monitored at least every month, and as needed on an ad hoc basis, to ensure the continuing safety of the participants. The Scientific Committee will determine the need to terminate the trial. Participant who experience any such event will be

For peer review only - http://bmjopen.bmj.com/site/about/guidelines.xhtml

directed to the appropriate hospital and all the necessary care will be ensured and followed-up until the participant has resolved or stabilized.

Ethics and dissemination

Ethical approval will be obtained from the ethics review committee of the Faculty of Medical Sciences, University of Sri Jayewardenepura and the University of Leicester, United Kingdom. Further permission for the proposed study will be obtained from the relevant authority at the Central Chest Clinic, Colombo. Sri Lanka.

Privacy and the confidentiality of all information and identities of participants will be strictly maintained and will not be disclosed when publishing the results of the study.

A copy of the test results obtained will be provided to each participant at the end of the trial for subsequent follow-up and treatment at the clinic. Compensation for travelling will be provided to all the participants. All study documents have been translated to Sinhala and Tamil to ensure clear communication. Participation will be without compulsion and each participant has the right to withdraw at any time, without providing a reason. Consent form and data sheets will be securely stored in a separate locked cupboard. Study computers will be password protected. All the data will be stored safely up to 6 years and after 6 years consent form and data sheets will be disposed of appropriately. Study team and IDMC only will have access to final trial dataset. Data from the Global RECHARGE Core Dataset will be made available following the completion of this project and we are considering the best tools to use to make this database available to the wider community. Any modifications of the protocol will be updated on trial registry (ISRCTN) and will be informed to the ethics review committee and the participants. Participants will be provided the provisions for re-consenting after any change of the approved protocol. It will be made clear in the publication of trial findings.

For peer review only

For peer review only - http://bmjopen.bmj.com/site/about/guidelines.xhtml

Author affiliations

[1]Faculty of Health Sciences, KAATSU International University, Sri Lanka
[2]Centre for Exercise and Rehabilitation Science, NIHR Leicester Biomedical Research Centre-Respiratory, University Hospitals of Leicester NHS Trust, UK
[3]Department of Respiratory Sciences, University of Leicester, Leicester, UK,
[4]Sports Medicine Unit, Colombo South Teaching Hospital, Kalubowila, Sri Lanka
[5]Department of Nursing and Midwifery, Faculty of Allied Health Sciences, University of Sri Jayewardenepura, Sri Lanka
[6] National Hospital of Sri Lanka and Central Chest Clinic, Colombo, Sri Lanka
[7]Primary Care Respiratory Group, Sri Lanka
[8]Faculty of Health, University of Plymouth, UK
[9]School of Sport, Exercise and Health Sciences, Loughborough University, UK
[10]Department of Economics, University of Sheffield, Sheffield, UK
[11]Faculty of Medical Sciences, University of Sri Jayewardenepura, Sri Lanka

Author contribution All authors of the paper have contributed to the conception and design of the study. ARJ drafted the manuscript. All authors of the paper have revised the content and approved the final version to be published. All authors are accountable for all aspects of the work.

Funding This research was funded by the National Institute for Health Research (NIHR) (17/63/20) using UK aid from the UK Government to support global health research. The views expressed in this publication are those of the author(s) and not necessarily those of the NIHR or the UK Department of Health and Social Care.

Competing interests None declared.

For peer review only - http://bmjopen.bmj.com/site/about/guidelines.xhtml

References

1. Singh D, Agusti A, Anzueto A, Barnes PJ, Bourbeau J, Celli BR, et al. Global Strategy for the Diagnosis, Management, and Prevention of Chronic Obstructive Lung Disease: the GOLD science committee report 2019. *Eur Respir J.* 2019;53(5).
2. Alwan A. Global Status Report on Non-Communicable Diseases. WHO, 2010
3. World Health Organization. Global surveillance, prevention and control of Chronic Respiratory Diseases, Switzerland, 2007; 22-23.
4. Amarasiri L, Fernando A, Gunasekara K, Gunasinghe W, Madegedara D, Wickramasinghe R. The prevalence of Chronic Obstructive Pulmonary Disease in Sri Lanka. *Europian Respiratory Journal*, 2017; 2017(50):1212.
5. World Health Organization. WHO strategy for prevention and control of chronic respiratory diseases, Genewa, 2017.
6. Yasaratne D, Dharmage SC. Letter from Sri Lanka. *Respirology*. 2020;25:339-41.
7. Pinnock H, Kendall M, Murray SA, Worth A, Levack P, Porter M, et al. Living and dying with severe chronic obstructive pulmonary disease: Multi-perspective longitudinal qualitative study. *BMJ*. 2011;342:268.
8. Singh SJ, Halpin DMG, Salvi S, Kirenga BJ, Mortimer K. Comment Exercise and pulmonary rehabilitation for people with chronic lung disease in LMICs : challenges and opportunities. Lancet Respir [Internet]. 2019;7(12):1002–4.
9. Nici L, Donner C, Wouters E, Zuwallack R, Ambrosino N, Bourbeau J, et al. American Thoracic Society Documents American Thoracic Society / European Respiratory Society Statement on Pulmonary Rehabilitation. *Am J Respir Crit Care Med*. 2006;173(May):1390-413.
10. Mccarthy B, Casey D, Devane D, Murphy K, Murphy E, Lacasse Y, et al. Pulmonary rehabilitation for chronic obstructive pulmonary disease (Review). *Cochrane Database Syst Rev.* 2015;2015(2).
11. Jones AW, Taylor A, Gowler H, Kelly NO, Ghosh S, Bridle C. Systematic review of interventions to improve patient uptake and completion of pulmonary rehabilitation in COPD. *ERJ Open Res.* 2017;3.

For peer review only - http://bmjopen.bmj.com/site/about/guidelines.xhtml

12. Chan AW, Tetzlaff JM, Altman DG, Laupacis A, Gøtzsche PC, Krleža-Jerić K, et al. Spirit 2013 statement: Defining standard protocol items for clinical trials. *Chinese J Evidence-Based Med*. 2013;13(12):1501–7.
13. Bolton CE, Bevan-Smith EF, Blakey JD, et al. British Thoracic Society guideline on pulmonary rehabilitation in adults: accredited by NICE. *Thorax*. 2013;68:ii1-ii30.
14. Ries AL, Bauldoff GS, Casaburi R, Mahler DA, Rochester CL, Herrerias C. Pulmonary Rehabilitation; Joint ACCP/AACVPR Evidence-Based Clinical Practice Guidelines. *Chest*. 2007;131(5):4S-42S.
15. Blackstock FC, Evans RA. Rehabilitation in lung diseases: 'Education' component of pulmonary rehabilitation. *Respirology*. 2019;24(9):863–70
16. Spencer LM, McKeough ZJ. Maintaining the benefits following pulmonary rehabilitation: Achievable or not? *Respirology*. 2019;24(9):909–15.
17. Armstrong M, Vogiatzis I. Personalized exercise training in chronic lung diseases. *Respirology*. 2019;24(9):854–62.
18. Revill SM, Morgan MDL, Singh SJ, Williams J, Hardman AE. The endurance shuttle walk : a new field test for the assessment of endurance capacity in chronic obstructive pulmonary disease. *Thorax*. 1999;54(2):213–22
19. Yasuda H, Yamaya M, Nakayama K, Ebihara S, Sasaki T, Okinaga S, et al. Increased Arterial Carboxyhemoglobin Concentrations in Chronic Obstructive Pulmonary Disease. *Am J Respir Crit Care Med*. 2005;171:1246–51.
20. Durnin JVGA, Womersley J. Body fat assessed from total body density and its estimation from skinfold thickness: measurements on 481 men and women aged from 16 to 72 Years. *Br J Nutr*. 1974;32:77–96.
21. Doña E, Olveira C, Palenque FJ, Porras N, Dorado A, Martín-valero R, et al. Body Composition Measurement in Bronchiectasis: Comparison between Bioelectrical Impedance Analysis, Skinfold Thickness Measurement, and Dual-Energy X-ray Absorptiometry before and after Pulmonary Rehabilitation. *J Acad Nutr Diet.* 2018;118(8):1464–73.
22. Lanerolle MD, Lanerolle P, Atukorala S, Silva A De. Enhancing the accuracy of bio impedance analysis in assessing total body water. *Int J Body Compos* Res. 2012;10(1):29–34.
23. Bestall JC, Paul EA, Garrod R, Garnham R, Jones PW, Wedzicha JA. Usefulness of the Medical Research Council (MRC) dyspnoea scale as a measure of disability in patients with

For peer review only

For peer review only - http://bmjopen.bmj.com/site/about/guidelines.xhtml

chronic obstructive pulmonary disease. *Thorax*. 1999;54:581–6.

24. Herdman M, Gudex C, Lloyd A, Janssen M, Kind P, Parkin D, et al. Development and preliminary testing of the new five-level version of EQ-5D (EQ-5D5L). *Quality of Life Research*. 2011;20(10): 1727-736.
25. Van der Molen T, Willemse BW, Schokker S, et al. Development, validity and responsiveness of the Clinical COPD Questionnaire. *Health Qual Life Outcomes.* 2003;1:13.
26. Papaioannou M, Pitsiou G, Manika K, Kontou P, Zarogoulidis P, Sichletidis L, Kioumis IP. COPD assessment test: A simple tool to evaluate disease severity and response to treatment. *J of Chronic Obstructive Pulmonary Disease*. 2014;11:489- 95.
27. Reilly MC, Zbrozek IAS, Dukes EM. The Validity and Reproducibility of a Work Productivity and Activity Impairment Instrument. *Pharmacoeconomics*. 1993;4(5):353–65.
28. Zigmond AS, Snaith RP. The hospital anxiety and depression scale. *Acta Psychiatrica Scandinavica.* 1983;67(6):361-70.
29. Weisman IM, Marciniuk D, Martinez FJ, Sciurba F, Sue D, Myers J, et al. American Thoracic Society / American College of Chest Physicians ATS / ACCP Statement on Cardiopulmonary Exercise Testing. *Am J Respir Crit Care Med*. 2003;167:211–77.
30. Singh SJ, Morgan MD, Scott S, Walters D, Hardman AE. Development of a shuttle walking test of disability in patients with chronic airways obstruction. *Thorax*. 1992;47(12):1019-24.
31. Mahler DA, Horowitz MB. Perception of breathlessness during exercise in patients with respiratory disease. *Med Sci Sports Exerc*. 1994;26:1078–1081.
32. Borg GA. Psychophysical bases of perceived exertion. *Med Sci Sports Exerc*. 1982;14:377–381
33. Jones SE, Kon SSC, Canavan JL, Patel MS, Clark AL, Nolan CM, et al. The five-repetition sit-to-stand test as a functional outcome measure in COPD. *Thorax*. 2013;68:1015–20.
34. Bassett DR, John D. Use of pedometers and accelerometers in clinical populations: validity and reliability issues. *Phys Ther Rev*. 2010;15:135–42.
35. Choi L, Liu Z, Matthews CE, Buchowski MS. Validation of accelerometer wear and nonwear time classification algorithm. *Med Sci Sports Exerc*. 2011;43:357–64.
36. Demeyer H, Burtin C, Van Remoortel H, Hornikx M, Langer D, Decramer M, et al. Standardizing the analysis of physical activity in patients with COPD following a pulmonary rehabilitation program. *Chest* 2014 Aug;146(2):318-327

For peer review only

For peer review only - http://bmjopen.bmj.com/site/about/guidelines.xhtml

37. Troiano RP, Berrigan D, Dodd KW, Mâsse LC, Tilert T, McDowell M. Physical activity in the United States measured by accelerometer. *Med Sci Sports Exerc* 2008 Jan;40(1):181-188.

38. Lucas AO, Gilles HM. Approaches to economic evaluation. *Short Textbook of Public Health Medicine for the Tropics.* 2002. CRC Press.

39. Harris PA, Taylor R, Minor BL, Elliott V, Fernandez M, Neal LO, et al. The REDCap consortium: Building an international community of software platform partners. *J Biomed Inform*. 2019;95.

40. Braun V, Clarke V. Using thematic analysis in psychology.*Qual Res Psychol*. 2006;3:77–101

41. Maguire M, Delahunt B. Doing a Thematic Analysis : A Practical, Step-by-Step Guide for Learning and Teaching Scholars. *AISHE-J*. 2017;8(3):3351–14.

For peer review only

For peer review only - http://bmjopen.bmj.com/site/about/guidelines.xhtml

Table 1: Primary outcome measures- Feasibility and operational experience assessment	
**Feasibility of patient recruitment**	**Data sources**
Feasibility of screening and recruiting participants	Interviews with the healthcare professionals, screening log
Suitability of the inclusion criteria	Interviews with the healthcare professionals, screening log
Number of eligible patients, number of patients screened, number of patients invited to take part, actual number of participants who consent to take part	Screening log
Number of patients who refuse, drop out and the reasons for refuse and drop out	Interviews with the patients, screening log
**Operational experience of intervention delivery**	
Service provider and multi-disciplinary teams' willingness and ability to deliver the PR	Interviews with healthcare professionals
The practicality of delivering the intervention in the proposed setting	Interviews with healthcare professionals and focus groups with participants
The time needed to collect the data<br>Baseline visit- Time taken for each measure (each individual questionnaire and physical measure)<br>Follow-up visit- Time taken for each measure (each individual questionnaire and physical measure)	Interviews with the healthcare professionals, Rehabilitation records
Data completeness and accuracy	Interviews with the healthcare professionals, Rehabilitation records, RedCap
Adherence to home exercise	Interviews with the patients and self-report exercise diary
The training and resources needed to deliver the intervention (ensuring readiness for a future much larger multi-center trial)	Interviews with the healthcare professionals and focus groups with participants, Rehabilitation records
Description of unintended events	Adverse events log, REDCap

For peer review only

Table 2: Secondary outcome measures		
**Outcome measures**	**Baseline**	**Post-intervention**
Socio-demographics	x	
Lung health (spirometry data, smoking status, number of COPD exacerbations in the last year)	x	
Comorbidities	x	
Treatments	x	
Nutritional status (Body Mass Index, mid upper arm circumference, skinfold thickness, self-report seven-day diet diary)	x	x
Disease burden (MRC dyspnea grade, CAT, CCQ)	x	x
Economic impact of disease (WPAI)	x	x
Quality of life (EQ-5D-5L)	x	x
Psychological wellbeing (Hospital Anxiety and Depression scale)	x	x
Physical function (5x sit-to-stand test)	x	x
Exercise capacity (ISWT, ESWT)	x	x
Accelerometer assessed physical activity (ActiGraph wGT3x-BT)	x	x

For peer review only

Table 3: Accelerometry data collection and processing parameters	
Accelerometer Model	ActiGraph wGT3X-BT (version 6.13.4; firmware 1.9.2)
Serial number range	Twenty unique devices will be used ranging from MOS2E09190601 to MOS2E24190146 averaging six deployments per device (same serial used for baseline and follow-up wear periods)
Piezosensor orientation	Triaxial
Mode setup	Mode 29 (x, y, z, steps, lux)
Original sample rate	100 Hz (.gt3x file format)
Deployment method	Baseline:<br>Fitted by research team on Day 0 (Baseline PR Assessment)<br>Fitted by participant on Day 1<br>Follow-up:<br>Fitted by research team Day 0 (11th/12th session)<br>Fitted by participant on Day 1
Location worn	Anterior hip adjacent to the mid-line of the thigh
Requested days of wear	7 days of free-living (Day 1-7; 10,080 epochs)
Initialization	Not deployed in delay mode in order to standardised capture of Day 0 (00:00) with stop time based on date of first PR class (baseline) and data of discharge assessment (follow-up)
Wear instructions	Wear continuously except for sleep and water based activities
Non-wear appropriation	≥60 min of consecutive 0s with allowance for 2 minutes of interruptions were deemed biologically implausible and coded as non-wear
Valid day criteria	≥8 hours of valid waking wear time
Valid file	≥4 valid days for each of the two time points
Missing data	Data modelling or imputation will not be performed
Epoch length	60 seconds
Intensity classification (absolute)	Uniaxial (x-axis) cut-points as follows: Sedentary time <100 cpm; Light intensity 100-2019 cpm; Moderate intensity 2020-5998cpm; Vigorous intensity ≥5999cpm (Moderate-to-vigorous intensity ≥2020 cpm)
Intensity classification (relative)	Uniaxial (x-axis) cut-points based on Endurance Shuttle Walk Test performance

For peer review only

Table 4: The variables used to calculate fixed and recurrent costs (not an exhaustive list)	
**Fixed costs**	**Recurrent costs**
Venue hire	Venue hire
Electrical equipment (laptop, printer, projector)	Staff time to conduct PR (assessment at baseline and discharge, conduct PR classes, telephone calls and data entry)
Equipment for PR (weights, treadmill, cycle ergometer, country-specific equipment, step-up box, chairs)	Disposable equipment (for blood glucose monitor, spirometer mouthpieces, nose-clips, glyceryl trinitrate spray)
Equipment for shuttle walking tests (cones, licences, stop watches, tape measure, electrical equipment to play audio)	Servicing costs (spirometer, PR equipment, specifically treadmills and cycle ergometers)
Equipment for PR assessment (height stadiometer, weight scales, sphygmomanometer, pulse oximeter, spirometer, calibration syringe, country-specific equipment)	Miscellaneous (Oxygen cylinders, questionnaire licences, stationery (paper))
Additional safety equipment (blood glucose monitor, Oxygen cylinder holder)	
Miscellaneous (filing cabinets, storage units, questionnaire translations, questionnaire licences, staff uniform)	

For peer review only

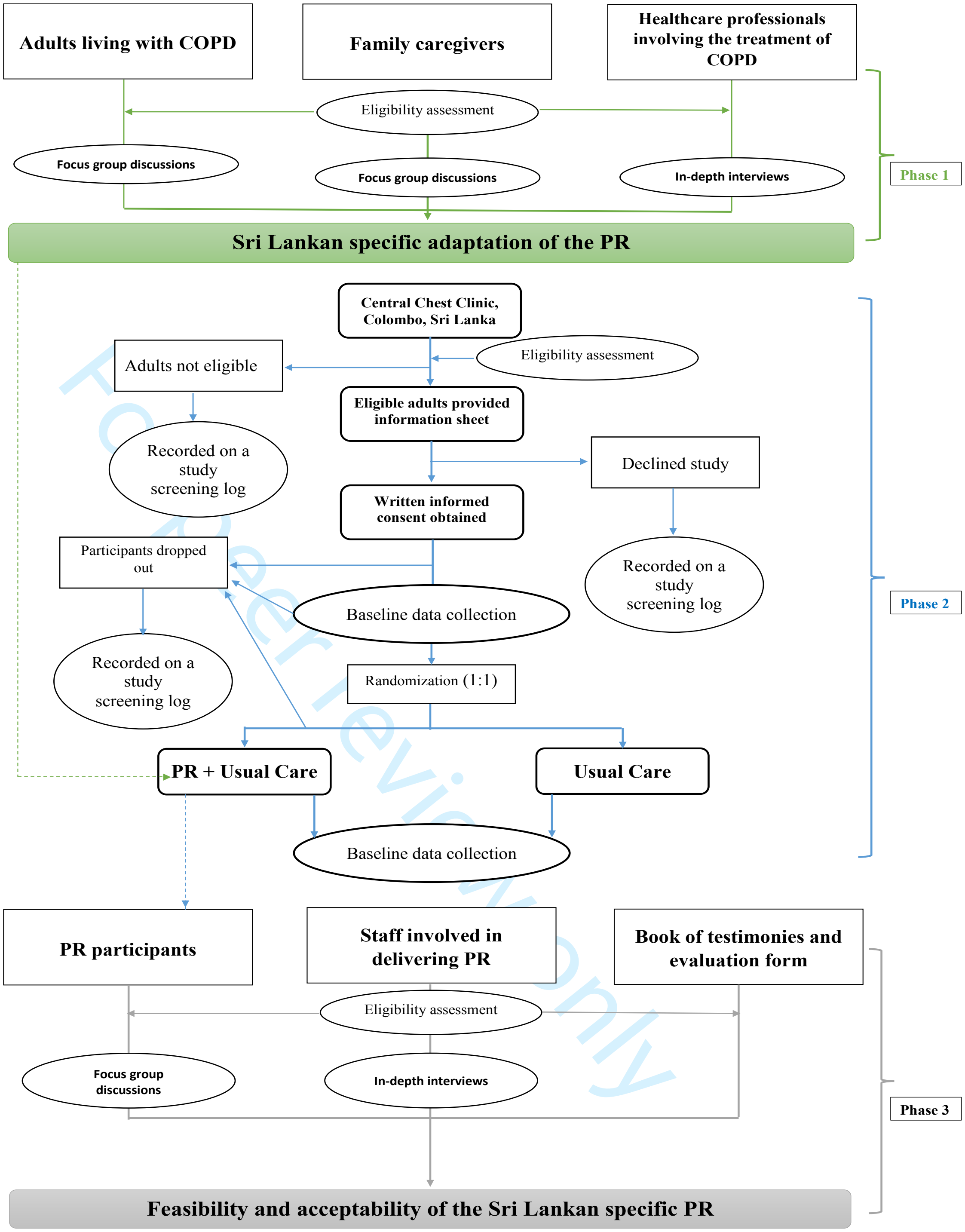

Figure 1: Flow of participant of the three phases of the study

Appendices

Information sheet

Cultural adaptation of Pulmonary Rehabilitation and subsequent testing in a Randomised Controlled Feasibility Trial for Adults with Chronic Obstructive Pulmonary Disease in Sri Lanka

I am Prof. S, W. Wimalasekera, of Department of Physiology, Faculty of Medical Sciences, University of Sri Jayewardenepura. I and my team of health care personnel are, conducting a research on "Testing the Feasibility and Acceptability of a Culturally Appropriate Hospital-Based Pulmonary Rehabilitation (PR) Programme for Adults with Chronic Obstructive Pulmonary Disease in Sri Lanka". I invite you to participate in the study. Please take time and read the following information carefully before giving your consent. You are free to ask questions to clarify facts which are not clear or ask for more information if you need.

Purpose of the study

The purpose of this study is to devise an appropriate PR programme and then determine the feasibility and acceptability of this programme for adults living with COPD in Sri Lanka and assess the potential for a future trial of its effectiveness.

When you participate you will be required to do the following

You will be requested to participate several components of the study.

You will participate in a group discussion conducted in a quiet room in the clinic (conference hall) with very minimal disturbances. At the beginning of the discussion you will answer some questions regards your personal information, (age, gender, history of the disease condition). Then the interviewer will ask some questions regarding your thoughts and ideas about pulmonary rehabilitation. You and your group members can freely comment on the questions or can add your ideas to the discussion. The interview takes around forty-five minutes of time.

You will also participate in a feasibility trial which you will be allocated to one of the two groups. One group will receive usual care and PR and the other group will receive only usual care which consists of pharmacological treatment and brief information about disease condition, medication and inhaler techniques. PR is typically a 6-week rolling programme and consisting of 12 sessions in total, delivered twice a week. Sessions usually last approximately two hours, with one hour for exercise training and one hour for education. Some routine and specific test and measurements will be taken before and after the intervention will describe any changes in the health of the adults living with COPD.

If you are allocated to the group who receive the PR, you will be asked to share your experiences regarding the PR and subjected modification and opinions on pulmonary rehabilitation.

In order to maintain accuracy, and to keep records, your discussion will be recorded by the research team members with your permission. Your participation in the project will remain confidential and the provision of your name and address is necessary for us to maintain records. Any personal information gathered during the study will not be disclosed to a third party. Your responses to the questions and will

For peer review only - http://bmjopen.bmj.com/site/about/guidelines.xhtml

only be used for the purpose of this project. You can be assured that you will remain anonymous if you take part in the project. Your participation in this project is entirely voluntary. You are not obliged to take part. If you do not wish to take part, you can withdraw without giving a reason and your medical care will not be affected, further you will not be contacted again. Similarly, if you do agree to participate, you are free to withdraw at any time during the project.

What are the possible benefits of taking part?

By participating in the study, you will contribute to develop a PR programme which helps to improve the quality of life of the adults living with COPD. As well as you will contribute to determine the feasibility and acceptability of this programme within the health care system in Sri Lanka.

What are the possible disadvantages and risks of taking part?

You will be undertaking some physical tests as part of the research. Therefore, there may be a very small risk of falls, breathlessness, changes in blood pressure and changes in heart rate. However, these risks are very rare, and trained staff and emergency equipment will be available to deal with any serious events. Participants who experience any such event will be directed to the appropriate hospital and all the necessary care will be ensured and followed-up until the participant has resolved or stabilized.

What will happen if you don't carry on with the study?

If you withdraw from the study all the information and data collected from you, to date, will be destroyed and your name would be removed from all the research study files. There will not be any loss or impact on your routine medical care which you are entitled to.

What will happen to the results of the research study?

The results of all the participants of the study will be used for scientific research. All data will be available only to the researchers and information provided will be strictly confidential. Your participation in the study is highly appreciated. Privacy and confidentiality of data gathered is completely secure and will be used only for research purposes. If you have any questions or any concerns with regards to the study and need further information you can call the telephone number listed below. Further you can send any complaint to the above postal address or email address.

Yours faithfully

Prof. S.W. Wimalasekera

Department of Physiology, Faculty of Medical Sciences, University of Sri Jayewardenepura

T.P No. 0777487203 e-mail : savithriww@yahoo.com

Ethics Review Committee – University of Sri Jayewardenepura - +94 11 2758000 Ext 4075e

For peer review only

Consent Form: **Randomised Controlled Feasibility Trial**

Cultural adaptation of Pulmonary Rehabilitation and subsequent testing in a Randomised Controlled Feasibility Trial for Adults with Chronic Obstructive Pulmonary Disease in Sri Lanka

1.Have you read the information sheet? (please keep a copy for yourself) YES/NO

2.Have you had an opportunity to discuss this and ask any questions? YES/NO

3. Have you had satisfactory answers to all your questions? YES/NO

4. Have you received enough information about the study? YES/NO

5. Who explained the study to you--

6. Do you understand that you are free to withdraw from the study at any time,

without having to give a reason YES/NO

7. All personal details will be treated as STRICTLY CONFIDENTIAL.

Do you give your permission for these individuals to have access to the records? YES/NO

8. Have you had sufficient time to come to your decision? YES/NO

9. Do you agree to take part in this study? YES/NO

Participant's signature --------------------------------------- Date------------------------

By the Witness

I have been present while the procedure was explained to the participant and I have witness his/ her willingness to take part in the study.

Signature of witness-- Date-------------------------------

(The witness should be a person not connected to the study)

Name ---

Contact details--

For peer review only

STANDARD PROTOCOL ITEMS: RECOMMENDATIONS FOR INTERVENTIONAL TRIALS

SPIRIT 2013 Checklist: Recommended items to address in a clinical trial protocol and related documents*

Section/item	Item No	Description	Page(s)
**Administrative information**			
Title	1	Descriptive title identifying the study design, population, interventions, and, if applicable, trial acronym	01
Trial registration	2a	Trial identifier and registry name. If not yet registered, name of intended registry	02
	2b	All items from the World Health Organization Trial Registration Data Set	01 - 19
Protocol version	3	Date and version identifier	NA
Funding	4	Sources and types of financial, material, and other support	19
Roles and responsibilities	5a	Names, affiliations, and roles of protocol contributors	01,18
	5b	Name and contact information for the trial sponsor	19
	5c	Role of study sponsor and funders, if any, in study design; collection, management, analysis, and interpretation of data; writing of the report; and the decision to submit the report for publication, including whether they will have ultimate authority over any of these activities	19
	5d	Composition, roles, and responsibilities of the coordinating centre, steering committee, endpoint adjudication committee, data management team, and other individuals or groups overseeing the trial, if applicable (see Item 21a for data monitoring committee)	17
**Introduction**			
Background and rationale	6a	Description of research question and justification for undertaking the trial, including summary of relevant studies (published and unpublished) examining benefits and harms for each intervention	03
	6b	Explanation for choice of comparators	NA
Objectives	7	Specific objectives or hypotheses	04
Trial design	8	Description of trial design including type of trial (eg, parallel group, crossover, factorial, single group), allocation ratio, and framework (eg, superiority, equivalence, noninferiority, exploratory)	04

For peer review only - http://bmjopen.bmj.com/site/about/guidelines.xhtml

Methods: Participants, interventions, and outcomes

Study setting	9	Description of study settings (eg, community clinic, academic hospital) and list of countries where data will be collected. Reference to where list of study sites can be obtained	05
Eligibility criteria	10	Inclusion and exclusion criteria for participants. If applicable, eligibility criteria for study centres and individuals who will perform the interventions (eg, surgeons, psychotherapists)	05
Interventions	11a	Interventions for each group with sufficient detail to allow replication, including how and when they will be administered	07
	11b	Criteria for discontinuing or modifying allocated interventions for a given trial participant (eg, drug dose change in response to harms, participant request, or improving/worsening disease)	17.18
	11c	Strategies to improve adherence to intervention protocols, and any procedures for monitoring adherence (eg, drug tablet return, laboratory tests)	09, 18
	11d	Relevant concomitant care and interventions that are permitted or prohibited during the trial	18
Outcomes	12	Primary, secondary, and other outcomes, including the specific measurement variable (eg, systolic blood pressure), analysis metric (eg, change from baseline, final value, time to event), method of aggregation (eg, median, proportion), and time point for each outcome. Explanation of the clinical relevance of chosen efficacy and harm outcomes is strongly recommended	08 - 10
Participant timeline	13	Time schedule of enrolment, interventions (including any run-ins and washouts), assessments, and visits for participants. A schematic diagram is highly recommended (see Figure)	06
Sample size	14	Estimated number of participants needed to achieve study objectives and how it was determined, including clinical and statistical assumptions supporting any sample size calculations	10
Recruitment	15	Strategies for achieving adequate participant enrolment to reach target sample size	07

Methods: Assignment of interventions (for controlled trials)

Allocation:

Sequence generation	16a	Method of generating the allocation sequence (eg, computer-generated random numbers), and list of any factors for stratification. To reduce predictability of a random sequence, details of any planned restriction (eg, blocking) should be provided in a separate document that is unavailable to those who enrol participants or assign interventions	10

For peer review only - http://bmjopen.bmj.com/site/about/guidelines.xhtml

Allocation concealment mechanism	16b	Mechanism of implementing the allocation sequence (eg, central telephone; sequentially numbered, opaque, sealed envelopes), describing any steps to conceal the sequence until interventions are assigned	10
Implementation	16c	Who will generate the allocation sequence, who will enrol participants, and who will assign participants to interventions	10
Blinding (masking)	17a	Who will be blinded after assignment to interventions (eg, trial participants, care providers, outcome assessors, data analysts), and how	10, 11
	17b	If blinded, circumstances under which unblinding is permissible, and procedure for revealing a participant's allocated intervention during the trial	NA
**Methods: Data collection, management, and analysis**			
Data collection methods	18a	Plans for assessment and collection of outcome, baseline, and other trial data, including any related processes to promote data quality (eg, duplicate measurements, training of assessors) and a description of study instruments (eg, questionnaires, laboratory tests) along with their reliability and validity, if known. Reference to where data collection forms can be found, if not in the protocol	11 - 15
	18b	Plans to promote participant retention and complete follow-up, including list of any outcome data to be collected for participants who discontinue or deviate from intervention protocols	07
Data management	19	Plans for data entry, coding, security, and storage, including any related processes to promote data quality (eg, double data entry; range checks for data values). Reference to where details of data management procedures can be found, if not in the protocol	17
Statistical methods	20a	Statistical methods for analysing primary and secondary outcomes. Reference to where other details of the statistical analysis plan can be found, if not in the protocol	17
	20b	Methods for any additional analyses (eg, subgroup and adjusted analyses)	17
	20c	Definition of analysis population relating to protocol non-adherence (eg, as randomised analysis), and any statistical methods to handle missing data (eg, multiple imputation)	17
**Methods: Monitoring**			
Data monitoring	21a	Composition of data monitoring committee (DMC); summary of its role and reporting structure; statement of whether it is independent from the sponsor and competing interests; and reference to where further details about its charter can be found, if not in the protocol. Alternatively, an explanation of why a DMC is not needed	17

	21b	Description of any interim analyses and stopping guidelines, including who will have access to these interim results and make the final decision to terminate the trial	17
Harms	22	Plans for collecting, assessing, reporting, and managing solicited and spontaneously reported adverse events and other unintended effects of trial interventions or trial conduct	17
Auditing	23	Frequency and procedures for auditing trial conduct, if any, and whether the process will be independent from investigators and the sponsor	17
**Ethics and dissemination**			
Research ethics approval	24	Plans for seeking research ethics committee/institutional review board (REC/IRB) approval	18
Protocol amendments	25	Plans for communicating important protocol modifications (eg, changes to eligibility criteria, outcomes, analyses) to relevant parties (eg, investigators, REC/IRBs, trial participants, trial registries, journals, regulators)	18
Consent or assent	26a	Who will obtain informed consent or assent from potential trial participants or authorised surrogates, and how (see Item 32)	7
	26b	Additional consent provisions for collection and use of participant data and biological specimens in ancillary studies, if applicable	NA
Confidentiality	27	How personal information about potential and enrolled participants will be collected, shared, and maintained in order to protect confidentiality before, during, and after the trial	18
Declaration of interests	28	Financial and other competing interests for principal investigators for the overall trial and each study site	19
Access to data	29	Statement of who will have access to the final trial dataset, and disclosure of contractual agreements that limit such access for investigators	18
Ancillary and post-trial care	30	Provisions, if any, for ancillary and post-trial care, and for compensation to those who suffer harm from trial participation	17,18
Dissemination policy	31a	Plans for investigators and sponsor to communicate trial results to participants, healthcare professionals, the public, and other relevant groups (eg, via publication, reporting in results databases, or other data sharing arrangements), including any publication restrictions	01
	31b	Authorship eligibility guidelines and any intended use of professional writers	19
	31c	Plans, if any, for granting public access to the full protocol, participant-level dataset, and statistical code	18

For peer review only

For peer review only - http://bmjopen.bmj.com/site/about/guidelines.xhtml

Appendices

Informed consent materials	32	Model consent form and other related documentation given to participants and authorised surrogates	See Appendix
Biological specimens	33	Plans for collection, laboratory evaluation, and storage of biological specimens for genetic or molecular analysis in the current trial and for future use in ancillary studies, if applicable	NA

*It is strongly recommended that this checklist be read in conjunction with the SPIRIT 2013 Explanation & Elaboration for important clarification on the items. Amendments to the protocol should be tracked and dated. The SPIRIT checklist is copyrighted by the SPIRIT Group under the Creative Commons "Attribution-NonCommercial-NoDerivs 3.0 Unported" license.

For peer review only

BMJ Open

Protocol for the Cultural Adaptation of Pulmonary Rehabilitation and Subsequent Testing in a Randomised Controlled Feasibility Trial for Adults with Chronic Obstructive Pulmonary Disease in Sri Lanka

Journal:	*BMJ Open*
Manuscript ID	bmjopen-2020-041677.R1
Article Type:	Protocol
Date Submitted by the Author:	26-Oct-2020
Complete List of Authors:	Jayamaha, Akila; KIU, Health Sciences<br>Perera, Chamilya; KIU, Health Sciences<br>Orme, Mark; University Hospitals of Leicester NHS Trust, Centre for Exercise and Rehabilitation Science, NIHR Leicester Biomedical Research Centre-Respiratory; University of Leicester, Department of Respiratory Sciences<br>Jones , Amy; University Hospitals of Leicester NHS Trust, Centre for Exercise and Rehabilitation Science, NIHR Leicester Biomedical Research Centre-Respiratory; University of Leicester, Department of Respiratory Sciences<br>Wijayasiri, Upendra; Colombo South Teaching Hospital, Sports Medicine Unit<br>Amarasekara, Thamara; University of Sri Jayewardenepura, Faculty of Allied Health Sciences<br>Karunatillake, Ravini ; National Hospital of Sri Lanka, Central Chest Clinic<br>Fernando, Amitha; National Hospital of Sri Lanka, Central Chest Clinic<br>Seneviratne, Anthony ; Primary Care Respiratory Group<br>Barton, Andy; University of Plymouth, Faculty of Health<br>Jones, Rupert; University of Plymouth, Faculty of Health<br>Yusuf, Zainab; Centre for Exercise and Rehabilitation Science, NIHR Leicester Biomedical Research Centre-Respiratory, University Hospitals of Leicester NHS Trust; University of Leicester, Department of Respiratory Sciences<br>Miah, Ruhme ; University of Leicester, Department of Respiratory Sciences; University Hospitals of Leicester NHS Trust, Centre for Exercise and Rehabilitation Science, NIHR Leicester Biomedical Research Centre-Respiratory<br>Malcolm, Dominic; Loughborough University, School of Sport, Exercise and Health Sciences<br>Matheson, Jesse; University of Sheffield, Department of Economics<br>Free, Robert; University of Leicester, Department of Respiratory Sciences,<br>Manise, Adrian ; University of Leicester, Department of Respiratory Sciences<br>Steiner, michael ; University of Leicester, Department of Respiratory Sciences; University Hospitals of Leicester NHS Trust, Centre for Exercise and Rehabilitation Science, NIHR Leicester Biomedical Research Centre-Respiratory

	Wimalasekera, Savithri; University of Sri Jayewardenepura, Faculty of Medical Sciences<br>Singh, Sally; University of Leicester, Department of Respiratory Sciences; University Hospitals of Leicester NHS Trust, Centre for Exercise and Rehabilitation Science, NIHR Leicester Biomedical Research Centre-Respiratory
**Primary Subject Heading**:	Respiratory medicine
Secondary Subject Heading:	Rehabilitation medicine
Keywords:	REHABILITATION MEDICINE, Chronic airways disease < THORACIC MEDICINE, Emphysema < THORACIC MEDICINE, Protocols & guidelines < HEALTH SERVICES ADMINISTRATION & MANAGEMENT

SCHOLARONE™
Manuscripts

I, the Submitting Author has the right to grant and does grant on behalf of all authors of the Work (as defined in the below author licence), an exclusive licence and/or a non-exclusive licence for contributions from authors who are: i) UK Crown employees; ii) where BMJ has agreed a CC-BY licence shall apply, and/or iii) in accordance with the terms applicable for US Federal Government officers or employees acting as part of their official duties; on a worldwide, perpetual, irrevocable, royalty-free basis to BMJ Publishing Group Ltd ("BMJ") its licensees and where the relevant Journal is co-owned by BMJ to the co-owners of the Journal, to publish the Work in this journal and any other BMJ products and to exploit all rights, as set out in our licence.

The Submitting Author accepts and understands that any supply made under these terms is made by BMJ to the Submitting Author unless you are acting as an employee on behalf of your employer or a postgraduate student of an affiliated institution which is paying any applicable article publishing charge ("APC") for Open Access articles. Where the Submitting Author wishes to make the Work available on an Open Access basis (and intends to pay the relevant APC), the terms of reuse of such Open Access shall be governed by a Creative Commons licence – details of these licences and which Creative Commons licence will apply to this Work are set out in our licence referred to above.

Other than as permitted in any relevant BMJ Author's Self Archiving Policies, I confirm this Work has not been accepted for publication elsewhere, is not being considered for publication elsewhere and does not duplicate material already published. I confirm all authors consent to publication of this Work and authorise the granting of this licence.

For peer review only

Protocol for the Cultural Adaptation of Pulmonary Rehabilitation and Subsequent Testing in a Randomised Controlled Feasibility Trial for Adults with Chronic Obstructive Pulmonary Disease in Sri Lanka

Akila R Jayamaha [1], Chamilya H Perera [1], Mark W Orme [2,3], Amy V Jones [2,3], Upendra K D C Wijayasiri [4], Thamara D Amarasekara [5], Ravini de S Karunatillake [6], Amitha C Fernando [6], Seneviratne A L P De S.[7], Andy Barton [8], Rupert Jones [8], Zainab K Yusuf [2,3], Ruhme B Miah [2,3], Dominic Malcolm [9], Jesse A Matheson [10], Robert C Free [3], Adrian Manise [3], Michael C Steiner [2,3] Savithri W Wimalasekera [11], Sally J Singh [2,3]

Correspondence to Akila Randika Jayamaha/1226arj@gmail.com/akila@kiu.ac.lk

For peer review only

Abstract

Introduction International guidelines recommend pulmonary rehabilitation (PR) should be offered to adults living with COPD, but there is limited PR available in Sri Lanka. Culturally appropriate PR needs to be designed and implemented in Sri Lanka. The study aims to adapt PR to the Sri Lankan context and determine the feasibility of conducting a future trial of the adapted PR in Sri Lanka.

Methods and analysis Phase-1: Develop the intervention: Focus groups with adults living with COPD, caregivers and nurses and in-depth interviews with doctors and physiotherapist will be conducted to inform PR adaptations. Phase-2: Randomised controlled feasibility trial will be conducted in Central Chest Clinic, Colombo, Sri Lanka. Eligible participants will be identified and will be invited to take part. A total of 50 participants will be recruited to the trial and randomised (1:1) into one of two groups; control group receiving usual care or the intervention group also receiving PR. The PR programme is likely to consist of 12 sessions of exercises and health education, delivered over 6 weeks. After completion of PR, routine measures in both groups will be assessed by a blinded assessor. The primary outcome measure is feasibility, including assessing eligibility, uptake and completion. Phase-3: Qualitative evaluation of the trail: Focus groups with participants and in-depth interviews with PR deliverers will be conducted to further determine feasibility and acceptability of PR, as well as ability to run a larger trial.

Ethics and dissemination Ethical approval was obtained from the ethics review committee of the Faculty of Medical Sciences, University of Sri Jayewardenepura, Sri Lanka and the

For peer review only - http://bmjopen.bmj.com/site/about/guidelines.xhtml

University of Leicester, UK. The results of the trial will be disseminated through patient and public involvement events, local and international conference proceedings, and peer-reviewed journals.

Trial registration: ISRCTN13367735

Keywords: COPD, Pulmonary Rehabilitation, Exercise, Education, Sri Lanka

Strengths and limitations

- This study is the first examining the feasibility and acceptability of a culturally appropriate pulmonary rehabilitation programme for adults with COPD in Sri Lanka.
- Taking a mixed-method approach, this study will provide a rich insight into delivering a trial of pulmonary rehabilitation in the Sri Lankan context.
- This study is a single center feasibility trial. As such, whilst findings will be an important first step in understanding the potential role of pulmonary rehabilitation in Sri Lanka and low- and middle-income countries more broadly, findings may not be generalizable to other regions of the world.

For peer review only

INTRODUCTION

Chronic obstructive pulmonary disease (COPD) is a progressive disease characterised by airflow obstruction and breathlessness. COPD is a major cause of morbidity and mortality throughout the world, corresponding to 6% of all deaths worldwide.[1] Further, more than 90% of COPD deaths occur in low and middle-income countries.[2] The most recent estimate of COPD prevalence in South Asia was 6.3% [3] with a prevalence in Sri Lanka of 10.5%,[4] similar to the estimated global COPD prevalence of 11.7%.[5] COPD is a significant burden to both patients and healthcare services.[6]

Chronic cough with sputum, breathlessness, physical inactivity and exercise intolerance resulting from dyspnoea or fatigue are common consequences of COPD.[1] Symptoms of COPD progressively worsen and people can become breathless, even at rest. Daily activities often become difficult as the condition worsens, impacting their quality of life.[7] The impact of COPD to the individual and to society makes the need for interventions to reverse the associated disability of paramount importance.

International guidelines recommend that pulmonary rehabilitation (PR) should be routinely offered to patients with chronic respiratory disease who have persistent symptoms, limited activity, and/or are unable to adjust to illness.[8,9] It is a low cost, high impact intervention that improves the quality of life, reduces suffering, reduces mortality and reduces economic loss, relieves dyspnoea and fatigue, improves exercise capacity, improves psychological and emotional function, and enhances an individual's self-management of their condition.[1] Having realised the benefits of PR in COPD, Western countries have incorporated this as an important structural component of healthcare delivery services.[10] Implementation of PR based as practiced in Western countries, in Sri Lanka requires adaptation to the local health service, population and culture. Despite its effectiveness, there is a significant need to understand the feasibility of conducting PR in low resource Sri Lankan health care setting and the acceptability of PR among Sri Lankan adults living with COPD and healthcare staff involved in its delivery. Uptake and completion of PR even in Western countries is an on-going challenge.[11] The need to maximize appeal of PR to patients and referrers is a global issue and one that is likely to be specific to a given location and population. There remains an unmet need for PR in Sri Lanka. To be

For peer review only

For peer review only - http://bmjopen.bmj.com/site/about/guidelines.xhtml

successful, PR must be not only evidence-based, but also designed and implemented in a manner sensitive to the context in which it is being delivered, such as culture and geography. Therefore, the aim of this study is to devise an appropriate PR programme and then determine the feasibility and acceptability of this programme for adults living with COPD in Sri Lanka and assess the potential for a future trial of its effectiveness.

The objectives of the study are to:

1. Explore the needs and perceptions of adults living with COPD, their care-givers /family members and health care professionals to inform the adaptations required for a PR programme suitable for the Sri Lankan context.
2. Determine the feasibility of conducting adapted hospital-based PR for people living with COPD.
3. Assess the acceptability of the PR among Sri Lankan adults living with COPD and healthcare staff involved in its delivery.
4. Describe any changes in health of the adults living with COPD following completion of PR.
5. Assess the feasibility of a future trial and estimate the required sample size.

METHODS AND ANALYSIS

Study design and registration

The proposed study will be conducted in three phases. Phase 1: A qualitative study will be conducted to inform the adaptations required to make PR specific to the Sri Lankan context. Phase 2 is a single blind randomised control feasibility trial. Adults living with COPD will be randomised (1:1) into adapted PR or usual care. Phase 3: Qualitative evaluation of the trial to determine the feasibility and acceptability of PR deliverers and participants. The trial will be conducted, analysed and reported according to the Standard Protocol Items: Recommendations for Interventional Trials (SPIRIT) statement [12] and trial has been registered on the ISRCTN website. Study was commenced upon ethical approval on 24/07/2020 for Phase 1 of the study. The design of the study and flow of participant enrolment is presented in figure 1.

For peer review only - http://bmjopen.bmj.com/site/about/guidelines.xhtml

Study setting

The study will be carried out at the Central Chest Clinic, Colombo, Sri Lanka which is a leading health care facility that provides treatment for adults with respiratory disease. A room allowing confidential discussions will be used for conducting focus group discussions among adults living with COPD, their caregivers /family members, and nurses. Semi structured interviews will be conducted among doctors and physiotherapists in a given quite room at the Central Chest Clinic as convenient to them and without interfering with their routine work. The venue for PR will be a large room at the Central Chest Clinic.

Participants

Key informants and suitable participants for phase 1 of the study will be identified by the researchers with the help of health care professionals involved in the treatment of COPD at Central Chest Clinic. Suitable participants will be purposively selected and informed verbally about the study by the researchers. After receiving a study information sheet, potential participants will be contacted to arrange an appointment, if they wish to take part. An opportunity to ask questions will be provided. If willing to take part in the study, they will be asked to provide written informed consent. Multi-religious and multi-ethnic Sri Lankan cultural diversity and gender will be considered when recruiting to the study. Adults living with COPD aged ≥18 years and Medical Research Council (MRC) dyspnoea score grade 2 or higher, Family member aged ≥18 years and looks after a patient with COPD and Health care professionals who have more than 1-year experience of managing patients with COPD and working in the government health care system of the country will be eligible to participate in the phase 1 of the study. Participants who provide written informed consent will be enrolled in the study.

After the adaptation of PR in phase 1, suitable participants for the Phase 2 randomised controlled feasibility trial will be identified by the Medical Officers in the Chest Clinic and will be invited to take part in an eligibility assessment. This assessment will determine eligibility for entry into the study. People eligible for inclusion in the trial will be: aged ≥18 years, will have a clinically confirmed diagnosis of COPD by a physician, confirmed COPD with spirometry based on GOLD criteria with $FEV_1/FVC<0.7$, and $FEV_1<80\%$ predicted, ≥1 exacerbation required a hospitalisation in the year preceding study, mMRC grade ≥2 and willing to provide informed consent. Adults with comorbidities such as severe or unstable cardiovascular, other internal

For peer review only

For peer review only - http://bmjopen.bmj.com/site/about/guidelines.xhtml

diseases and locomotor difficulties that preclude the exercise or malignant disease or other serious illness which will interfere with participation in the PR, will be excluded from the study. Individuals not eligible for the study will be recorded on a study screening log.

[insert figure 1 here]

Procedure

Eligible participants will be informed verbally about the study by the Medical Officers of the Central Chest Clinic. After receiving a study information sheet, potential participants will be contacted to arrange an appointment, if they wish to take part. An opportunity to ask questions will be provided. If willing to take part in the study, they will be asked to provide written informed consent. Information regarding the interest of participation in the study will be taken as field notes. Baseline outcome measures will be assessed. After baseline data collection, participants will be randomised (1:1) into two groups. One group will receive usual care and PR and the other group will receive only usual care which consists of pharmacological treatment and brief information about disease condition, medication and inhaler techniques. Experiences of the participants and PR deliverers regarding the acceptability and feasibility of PR will be explored in interviews and focus groups during Phase 3 of the study. Participants who did not complete the PR will be asked to take part in a drop-out interview and information provided freely by the participants will be collected as field notes.

Trial interventions

Intervention: Adapted pulmonary rehabilitation

Sri-Lankan specific PR will comprise the core elements of an evidence based rehabilitation, a programme of exercises [13, 14] and health education.[13, 14, 15] The detail of delivery and adaptations of PR will be informed by findings of the Phase 1 of the study. PR is typically a 6-week rolling programme that consists of 12 sessions in total.[13, 14] Sessions usually last approximately two hours, with one hour for exercise training and one hour for education. All the education sessions and the instructions during PR will be provided in Sinhala language as convenient to the participants. The duration of the PR trial and frequency and duration of sessions will be

For peer review only - http://bmjopen.bmj.com/site/about/guidelines.xhtml

discussed during Phase 1. All staff delivering PR will be trained and assisted by a nursing officer, a clinical nutritionist, an exercise physician and a physiotherapist. The venue for PR will be at the Central Chest Clinic, Colombo, Sri Lanka. The Central Chest Clinic will have a maximum capacity of 10 patients per PR class. The equipment required will be simple and include chairs, weights, and simple exercise equipment based on local availability, suitability and informed by qualitative work.[16]

In keeping with evidence-based guidelines [13, 14], discussions in Phase 1 will include the core exercise component of PR, notably resistance training (e.g. upper and lower limbs) and aerobic training (walking, sit-to-stand, step-ups) using minimal equipment and education. Each participant's exercise regimen will be individually prescribed and progressed [17] with walking speed will be prescribed using the ISWT.[18] The process in which this is achieved by patients will be informed by Phase 1. The education component will be delivered by an interdisciplinary team and will be informed by qualitative findings from Phase 1 with discussions facilitated by usual education topics as described in guidelines.[13, 14]

Usual care

Usual care will consist of pharmacological treatment and optimization, prescribed to patients after consultation with Medical Officers at the Central Chest Clinic. Brief information about disease condition, medication and inhaler techniques will be provided by Medical Officers and Nurses. Pulmonary rehabilitation or any form of exercise recommendation is not part of usual care.

Outcomes

Primary outcome

The primary outcome of the trial will be the feasibility and the acceptability of the PR intervention.

Feasibility

For peer review only - http://bmjopen.bmj.com/site/about/guidelines.xhtml

Measures to assess feasibility are provided in table 1 and include the comprehensive assessment of the feasibility of patient recruitment and the intervention delivery.

Table 1: Primary outcome measures- Feasibility and operational experience assessment

**Feasibility of patient recruitment**	**Data sources**
Feasibility of screening and recruiting participants	Interviews with the healthcare professionals, screening log
Suitability of the inclusion criteria	Interviews with the healthcare professionals, screening log
Number of eligible patients, number of patients screened, number of patients invited to take part, actual number of participants who consent to take part	Screening log
Number of patients who refuse, drop out and the reasons for refuse and drop out	Interviews with the patients, screening log
**Operational experience of intervention delivery**	
Service provider and multi-disciplinary teams' willingness and ability to deliver the PR	Interviews with healthcare professionals
The practicality of delivering the intervention in the proposed setting	Interviews with healthcare professionals and focus groups with participants
The time needed to collect the data<br>Baseline visit- Time taken for each measure (each individual questionnaire and physical measure)<br>Follow-up visit- Time taken for each measure (each individual questionnaire and physical measure)	Interviews with the healthcare professionals, Rehabilitation records
Data completeness and accuracy	Interviews with the healthcare professionals, Rehabilitation records, RedCap
Adherence to home exercise	Interviews with the patients and self-report exercise diary
The training and resources needed to deliver the intervention (ensuring readiness for a future much larger multi-center trial)	Interviews with the healthcare professionals and focus groups with participants, Rehabilitation records
Description of unintended events	Adverse events log, REDCap

Acceptability

The acceptability of the PR intervention among adults living with COPD and healthcare staff involved in its delivery will be assessed. Participants' experience of the PR, including any perceived benefits, challenges and changes they would make to the programme, will be explored in qualitative interviews and focus groups after their discharge assessment or withdrawal. The experience of healthcare professionals regarding the PR intervention, such as their confidence in delivering the programme, the components of PR, structure of PR, the patient adherence to the

For peer review only - http://bmjopen.bmj.com/site/about/guidelines.xhtml

PR exercises and how their perceptions changed over the course of the trial, insights into barriers and facilitators to referral, uptake and completion of PR ((i) attending at least 10 out of 12 designated PR sessions and (ii) attending the follow-up evaluation) will be explored in qualitative interviews at the end of the trial.

Secondary outcomes

The secondary outcomes of this study are provided in table 2. Comparison of secondary outcome measures of baseline and post intervention, will describe any changes in the health of the adults living with COPD following completion of PR

Table 2: Secondary outcome measures

Outcome measures	Baseline	Post-intervention
Socio-demographics	x	
Lung health (spirometry data, smoking status, number of COPD exacerbations in the last year)	x	
Comorbidities	x	
Treatments	x	
Nutritional status (Body Mass Index, mid upper arm circumference, skinfold thickness, self-report seven-day diet diary)	x	x
Disease burden (MRC dyspnea grade, CAT, CCQ)	x	x
Economic impact of disease (WPAI)	x	x
Quality of life (EQ-5D-5L)	x	x
Pittsburgh Sleep Quality Index (PSQI)	x	x
Psychological wellbeing (Hospital Anxiety and Depression scale)	x	x
Physical function (5x sit-to-stand test)	x	x
Exercise capacity (ISWT, ESWT)	x	x
International Physical Activity Questionnaire (IPAQ)	x	x
Accelerometer assessed physical activity (ActiGraph wGT3x-BT)	x	x

MRC - Medical Research Council, CAT- COPD Assessment Test, CCQ - Clinical COPD Questionnaire, WPAI – Work Productivity and Activity Impairment questionnaire, EQ-5D-5L - EuroQol Five Dimensions Five Levels, ISWT - Incremental Shuttle Walk Test, ESWT - Endurance Shuttle Walk Tests

Sample size and recruitment target

This study is a feasibility trial that aims to provide data for an accurate estimation of the required sample size for future trials. Therefore, a formal sample size calculation is not required. We aim to recruit and randomise 50 participants to the study (25 in each group).

For peer review only - http://bmjopen.bmj.com/site/about/guidelines.xhtml

Patient allocation, concealment, and blinding

Randomisation will occur through random permuted blocks to either the intervention group (PR+ usual care) or control group (usual care) in ratio 1:1 using Sealed envelope™.

The computer-generated patient allocation sequence will be monitored by an individual who is independent of the research team and will inform the research team of group allocations via telephone. Participants will be informed about their group allocation after providing informed consent and completing baseline assessments.

It will not be possible to blind patients to their group allocation due to the nature of PR. Research staff will be blinded to outcome measures. Participants will be advised not to reveal their group during the follow-up assessment. Any episodes of un-blinding will be documented and reported.

Data collection

Phase 1: Qualitative assessment for adaptation of PR

Focus groups with patient and family caregivers

Focus groups with adults living with COPD and separate focus groups with their family members/ caregivers will be conducted until data saturation. We anticipate conducting up to 5 focus groups with 6-8 participants in each. Data will be collected during patients' clinic visits. Focus group discussions will be audio-recorded, expected to last approximately 45-90 minutes, and will be conducted face-to-face by an interviewer and note-taker (observer). Focus groups will be transcribed verbatim, with identifiable information removed. Envisaged outcomes of the focus groups will include Sri Lankan specific adaptation of PR.

Interviews with healthcare professionals involving the treatment of COPD

Up to 15 in-depth interviews with healthcare professionals will be conducted until data saturation. Structured interviews will be conducted with healthcare professionals as convenient to them without interfering with their routine work. Interviews will be audio-recorded and will be conducted face-to-face by an interviewer. Interviews will be transcribed verbatim, with

For peer review only - http://bmjopen.bmj.com/site/about/guidelines.xhtml

identifiable information removed. Envisaged outcomes of the in-depth interviews will be a suitable design and content of PR acceptable respiratory health care deliverers in Sri Lanka.

Phase 2: Single blind randomised control feasibility trial

Data will be collected by trained researchers, following standard operating procedures during participants' clinic visits. Baseline and post intervention assessments will be carried out by the blinded Medical Officers consulting at the study setting.

Phase 3: Qualitative evaluation of the PR intervention

Focus groups with patients

Participants allocated to the intervention group will be invited to participate in focus group discussions at the end of their PR programme. Focus groups will give an insight on views, experiences, opinions and recommendations which will inform future PR programmes. We anticipate conducting up to 5 focus groups until data saturation. Each focus group discussion will be conducted with 6-8 participants in each.

Focus group discussions will be audio-recorded, expected to last approximately 45-90 minutes, and will be conducted by a trained moderator and a note-taker). Focus groups will be transcribed verbatim, with identifiable information removed. Consent will be obtained from participants prior to their involvement in focus groups.

Interviews with PR staff

Health care personnel involved in delivering PR will be invited to participate in in-depth interviews at the end of the study to discuss aspects of feasibility and acceptability, such as insights into barriers and facilitators to attendance, logistical barriers of running a PR programme and their perceptions, confidence of programme delivery and patients' experiences of the intervention. Details regarding previous experience on PR and prior training regarding PR will be assessed using brief questionnaire before commencing the in-depth interviews. We anticipate conducting up to 15 interviews, each expected to last approximately 15-60 minutes. Interviews

For peer review only - http://bmjopen.bmj.com/site/about/guidelines.xhtml

will be audio-recorded and will be conducted face-to-face by a trained interviewer. Interviews will be transcribed verbatim, with identifiable information removed.

Book of testimonies and evaluation form

Participants within PR will be asked to log their experience of PR as they progress through the programme. This will be in the form of a PR log book accessible to participants before, during and after sessions, as well as a dedicated evaluation form which will be provided as supplementary material. Staff involved in PR will also receive the same evaluation form at the end of the study.

Sample characteristics

Basic demographics including age, sex, religion, nationality, marital status, age of leaving full-time education, education level, ethnicity, employment status, monthly income, lung health, smoking status (packs per year), biomass fuel exposure, primary respiratory diagnosis, time since diagnosis in years, secondary respiratory diagnoses, family history of lung disease, comorbidities will be recorded at baseline.

Lung function

Spirometry (post bronchodilator Forced Expiratory Volume in the first second (FEV_1), post bronchodilator Forced Vital Capacity (FVC), FEV_1/FVC ratio, carboxyhemoglobin test [19], hospitalisations within the last 12 months, number of COPD exacerbations within the last 12 months, treatments will be collected as the baseline data prior to the randomisation of participants.

Nutritional status

Height (Holtain stadiometer), weight (calibrated weight scale) will be measured and body mass index will be calculated. Mid upper arm circumference of the participants will be measured using a measuring tape and skinfold thickness of seven sites (triceps, mid axillary, subscapular, chest, abdominal, supra iliac and thigh measurements) of the body will be measured using a validated caliper. Triplicated measurements will be taken by the same investigator and mean value will be

For peer review only - http://bmjopen.bmj.com/site/about/guidelines.xhtml

used for the calculation of body fat mass. Fat free mass will be calculated using Durnin and Womersley formula.[20, 21] Body composition analysis will be assessed using bioelectrical impedance (SFB7 Impedi Med dual frequency instrument, Impedi Med Limited, Australia) and software version 5.2.4.0.[22] Seven-day diet diary method will be used to assess the dietary history and calorie intake.

Disease burden

Breathlessness will be measured using MRC dyspnoea grade (5-items).[23] The health-related quality of life related to mobility, self-care, usual activities, pain/discomfort and anxiety-depression will be measured using EQ-5D-5L (25-items),[24] Clinical status of the airways, functional limitations and psychosocial dysfunction will be determined using CCQ (10 -items),[25] CAT (8-items) will be used to determine the severity of the COPD,[26] Modified brief pain inventory (3-items) will determine the commonly reported chest pain, WPAI (8-items) will be used to measure the effect of severity of COPD and general health on work productivity and regular activities [27] and HADS (14-items) will be used to measure the patient's emotional state and the presence or absence of clinically significant anxiety and depression.[28] Sleep quality of the participants will be measured using Pittsburgh Sleep Quality Index (PSQI) [29]. Disease burden will be measured as baseline data prior to the randomisation and post-intervention data.

Exercise capacity

Following a safety assessment for absolute and relative contraindications for field walking tests[30], maximal exercise capacity will be assessed using the incremental shuttle walking test (ISWT) [31] and endurance shuttle walking test (ESWT).[18] The ISWT and ESWT also include a pre-test and post-test measure of SpO_2, blood pressure, Borg rating of breathlessness [32], heart rate, Borg rate of perceived exertion.[33] The sit-to stand test will be completed for five continuous repetitions, to measure lower limb movement and strength.[34] All measures of exercise capacity will be measured at baseline prior to the randomisation and post-intervention.

Physical activity

Subjective health–related physical activity will be measured with the International Physical Activity Questionnaire (IPAQ) [35] at baseline prior to the randomisation and post-intervention.

For peer review only - http://bmjopen.bmj.com/site/about/guidelines.xhtml

319 Participants will be asked to wear an activity monitor (ActiGraph wGT3X-BT, ActiGraph, Pensacola, FL, USA), able to detect a range of PA intensities [36], on the right anterior hip during waking hours for seven days prior to attending PR (baseline) and for the seven consecutive days prior to their discharge assessment (follow-up); taking it off for water-based activities and sleep. The collection of physical activity data will coincide with a 7-day diet diary. Printed instructions will be provided to the participants regarding how to wear the monitor.

Full accelerometry methodology is provided in Table 3. Data will be processed as 60-s epoch files in ActiLife version 6.13.4 (ActiGraph, Pensacola, FL, USA). Non-wear will be defined as 60-minutes of consecutive zero counts with allowance for 2-minutes of interruptions.[37] Adherence to wearing the monitors will be assessed by examining the proportion of participants providing valid accelerometer data across a range of valid days (≥1-7 days) and minimum wear time (≥1-12 hours) thresholds. For reported group averages, only participants providing ≥4 valid days of ≥8 hours, for both time points will be included in the analyses.[38] Step count, time spent in different absolute intensity classifications of physical behaviours [39] and average movement intensity (activity counts per minute) will be reported. Time spent in physical activity matching participants' prescribed walking exercise intensity will be derived by aligning the average walking speed during the ESWT with the activity monitor counts per minute.

Table 3: Accelerometry data collection and processing parameters

Accelerometer Model	ActiGraph wGT3X-BT (version 6.13.4; firmware 1.9.2)
Serial number range	Twenty unique devices will be used ranging from MOS2E09190601 to MOS2E24190146 averaging six deployments per device (same serial used for baseline and follow-up wear periods)
Piezosensor orientation	Triaxial
Mode setup	Mode 29 (x, y, z, steps, lux)
Original sample rate	100 Hz (.gt3x file format)
Deployment method	Baseline:<br>Fitted by research team on Day 0 (Baseline PR Assessment)<br>Fitted by participant on Day 1<br>Follow-up:<br>Fitted by research team Day 0 ($11^{th}/12^{th}$ session)<br>Fitted by participant on Day 1
Location worn	Anterior hip adjacent to the mid-line of the thigh
Requested days of wear	7 days of free-living (Day 1-7; 10,080 epochs)

For peer review only - http://bmjopen.bmj.com/site/about/guidelines.xhtml

Initialization	Not deployed in delay mode in order to standardised capture of Day 0 (00:00) with stop time based on date of first PR class (baseline) and data of discharge assessment (follow-up)
Wear instructions	Wear continuously except for sleep and water based activities
Non-wear appropriation	≥60 min of consecutive 0s with allowance for 2 minutes of interruptions were deemed biologically implausible and coded as non-wear
Valid day criteria	≥8 hours of valid waking wear time
Valid file	≥4 valid days for each of the two time points
Missing data	Data modelling or imputation will not be performed
Epoch length	60 seconds
Intensity classification (absolute)	Uniaxial (x-axis) cut-points as follows: Sedentary time <100 cpm; Light intensity 100-2019 cpm; Moderate intensity 2020-5998cpm; Vigorous intensity ≥5999cpm (Moderate-to-vigorous intensity ≥2020 cpm)
Intensity classification (relative)	Uniaxial (x-axis) cut-points based on Endurance Shuttle Walk Test performance

Patient and Public Involvement

Adults living with COPD often tell us how having COPD impacts their lives and that they often find it challenging to know what they can do to better manage their condition. When they talk about their self-management strategies, it is clear that education and exercise support would be of value. Also, they revealed the necessity of a programme to support their condition and willingness to attend such a programme. Patients are generally positive about being able to access support but there is little available to them. The study was planned to fulfill the need of adults living with COPD. The delivery and adaptations of the trial intervention (Sri-Lankan specific PR) will also be informed by the adults living with COPD, their care-givers /family members and health care professionals. Priorities, experience, and preferences of the stakeholders will be utilized to design the PR. Health care professionals involved in the treatment of COPD at Central Chest Clinic will be involved in the recruitment as key informants and conduct of the study. The feasibility and acceptability of the PR intervention among adults living with COPD and healthcare staff involved in its delivery will be assessed in qualitative interviews at the end of the trial. The results of the trial will be disseminated through patient and public involvement events, local and international conference proceedings. As well as all the research

For peer review only - http://bmjopen.bmj.com/site/about/guidelines.xhtml

participants, stakeholders and individuals with COPD will be openly invited to take part in an event organized at the central chest clinic to reveal the study findings.

Costing

As part of this trial, the cost of creating a Sri-Lankan adapted PR programme will be calculated. This will include both single and recurrent costs. Single payments include the necessary costs to set up and run a programme, whilst recurrent cost refers to any item with a life expectancy of 1 year or less and typically includes disposable materials.[40] The fixed costs will be captured prior to the first participant enrolling into the programme and the recurrent costs will be collected at the mid-stage of recruitment. An average cost per participant will be calculated. Table 4 demonstrates the variables that will be used to calculate fixed and recurrent costs.

Table 4: The variables used to calculate fixed and recurrent costs (not an exhaustive list)

**Fixed costs**	**Recurrent costs**
Venue hire	Venue hire
Electrical equipment (laptop, printer, projector)	Staff time to conduct PR (assessment at baseline and discharge, conduct PR classes, telephone calls and data entry)
Equipment for PR (weights, treadmill, cycle ergometer, country-specific equipment, step-up box, chairs)	Disposable equipment (for blood glucose monitor, spirometer mouthpieces, nose-clips, glyceryl trinitrate spray)
Equipment for shuttle walking tests (cones, licences, stop watches, tape measure, electrical equipment to play audio)	Servicing costs (spirometer, PR equipment, specifically treadmills and cycle ergometers)
Equipment for PR assessment (height stadiometer, weight scales, sphygmomanometer, pulse oximeter, spirometer, calibration syringe, country-specific equipment)	Miscellaneous (Oxygen cylinders, questionnaire licences, stationery (paper))
Additional safety equipment (blood glucose monitor, Oxygen cylinder holder)	

For peer review only - http://bmjopen.bmj.com/site/about/guidelines.xhtml

Miscellaneous (filing cabinets, storage units, questionnaire translations, questionnaire licences, staff uniform)	

Data management

Data collected during the study will be entered into a database using Research Electronic Data Capture (REDCap), which is a web-based platform.[41] Access to the database will be via a secure password protected web-interface. The participants will be identified by a study-specific identification code. Data will be validated using real-time data entry validation and electronic checks lead by the Independent Data Monitoring Committee (IDMC), established at the University of Leicester, UK.

Quantitative data analysis

The data will be analyzed using IBM SPSS Statistics for Windows, Version 23.0. Data for baseline and follow-up time-points will be presented as descriptive statistics as appropriate. No inferential statistics will be performed due to the feasibility design of the trial.

Qualitative data analysis

Qualitative data will be analyzed using Thematic Analysis. This approach follows six distinct stages: familiarization with data; generating initial codes; searching for themes; reviewing themes; defining and naming themes and producing the report.[42] The responsible investigator will carry out initial coding and a sample of focus group transcripts will be coded by a second member of the team to improve consistency and to enhance interpretive authenticity. Throughout the data analysis, the team will meet to discuss and review emerging themes and search the accounts that provide contesting views of the same phenomena. Close attention will be paid to the complexity and interactions inherent in the focus group data.[43]

Adverse events

For peer review only - http://bmjopen.bmj.com/site/about/guidelines.xhtml

All adverse events and serious adverse events will be recorded on an adverse event log, within study trial management paperwork, case report forms and REDCap. There will be no formal interim analysis of data due to the feasibility nature of the trial. The IDMC will review high level safety data. Adverse events will be monitored at least every month, and as needed on an ad hoc basis, to ensure the continuing safety of the participants. The Scientific Committee will determine the need to terminate the trial. Participants who experience any such event will be directed to the appropriate hospital and all the necessary care will be ensured and followed-up until the participant has resolved or stabilized.

Ethics and dissemination

Ethical approval was obtained from the ethics review committee of the Faculty of Medical Sciences, University of Sri Jayewardenepura (FMS/USJP ERC 64/19) and the University of Leicester, United Kingdom (26770). Further permission for the proposed study will be obtained from the relevant authority at the Central Chest Clinic, Colombo. Sri Lanka. Privacy and the confidentiality of all information and identities of participants will be strictly maintained and will not be disclosed when publishing the results of the study. A copy of the test results obtained will be provided to each participant at the end of the trial for subsequent follow-up and treatment at the clinic. Compensation for travelling will be provided to all the participants. All study documents will be translated to Sinhala and Tamil to ensure clear communication. Participation will be without compulsion and each participant has the right to withdraw at any time, without providing a reason. Consent form and data sheets will be securely stored in a separate locked cupboard. Study computers will be password protected. The participants (adults living with COPD) of the phase 1 study also will be provided the opportunity to participate the PR. All the data will be stored safely up to 6 years and after 6 years consent form and data sheets will be disposed of appropriately. Study team and IDMC only will have access to final trial dataset. Data from the Global RECHARGE Core Dataset will be made available following the completion of this project and we are considering the best tools to use to make this database available to the wider community. Any modifications of the protocol will be updated on trial registry (ISRCTN) and will be informed to the ethics review committee and the participants. Participants will be

For peer review only

For peer review only - http://bmjopen.bmj.com/site/about/guidelines.xhtml

provided the provisions for re-consenting after any change of the approved protocol. It will be made clear in the publication of trial findings.

Author affiliations

[1]Faculty of Health Sciences, KAATSU International University, Sri Lanka
[2]Centre for Exercise and Rehabilitation Science, NIHR Leicester Biomedical Research Centre-Respiratory, University Hospitals of Leicester NHS Trust, UK
[3]Department of Respiratory Sciences, University of Leicester, Leicester, UK,
[4]Sports Medicine Unit, Colombo South Teaching Hospital, Kalubowila, Sri Lanka
[5]Department of Nursing and Midwifery, Faculty of Allied Health Sciences, University of Sri Jayewardenepura, Sri Lanka
[6] National Hospital of Sri Lanka and Central Chest Clinic, Colombo, Sri Lanka
[7]Primary Care Respiratory Group, Sri Lanka
[8]Faculty of Health, University of Plymouth, UK
[9]School of Sport, Exercise and Health Sciences, Loughborough University, UK
[10]Department of Economics, University of Sheffield, Sheffield, UK
[11]Faculty of Medical Sciences, University of Sri Jayewardenepura, Sri Lanka

Author contribution

All authors (Akila R Jayamaha, Chamilya H Perera, Mark W Orme, Amy V Jones, Upendra K D C Wijayasiri, Thamara D Amarasekara, Ravini de S Karunatillake, Amitha C Fernando, Seneviratne A L P De S., Andy Barton, Rupert Jones, Zainab K Yusuf, Ruhme B Miah, Dominic Malcolm, Jesse A Matheson, Robert C Free, Adrian Manise, Michael C Steiner, Savithri W Wimalasekera, Sally J Singh) have substantially contributed to the conception and design of the study. Akila R Jayamaha drafted the manuscript. All authors of the paper have revised the

For peer review only

For peer review only - http://bmjopen.bmj.com/site/about/guidelines.xhtml

content and approved the final version to be published. All authors are accountable for all aspects of the work.

Funding This research was funded by the National Institute for Health Research (NIHR) (17/63/20) using UK aid from the UK Government to support global health research. The views expressed in this publication are those of the author(s) and not necessarily those of the NIHR or the UK Department of Health and Social Care.

Competing interests None declared.

[insert figure caption]

Figure 1: Flow of participant of the three phases of the study

References

1. Singh D, Agusti A, Anzueto A, Barnes PJ, Bourbeau J, Celli BR, et al. Global Strategy for the Diagnosis, Management, and Prevention of Chronic Obstructive Lung Disease: the GOLD science committee report 2019. *Eur Respir J.* 2019;53(5).
2. Alwan A. Global Status Report on Non-Communicable Diseases. WHO, 2010
3. World Health Organization. Global surveillance, prevention and control of Chronic Respiratory Diseases, Switzerland, 2007; 22-23.
4. Amarasiri L, Fernando A, Gunasekara K, Gunasinghe W, Madegedara D, Wickramasinghe R. The prevalence of Chronic Obstructive Pulmonary Disease in Sri Lanka. *Europian Respiratory Journal*, 2017; 2017(50):1212.
5. World Health Organization. WHO strategy for prevention and control of chronic respiratory diseases, Genewa, 2017.
6. Yasaratne D, Dharmage SC. Letter from Sri Lanka. *Respirology*. 2020;25:339-41.
7. Pinnock H, Kendall M, Murray SA, Worth A, Levack P, Porter M, et al. Living and dying with severe chronic obstructive pulmonary disease: Multi-perspective longitudinal qualitative study. *BMJ*. 2011;342:268.

For peer review only - http://bmjopen.bmj.com/site/about/guidelines.xhtml

8. Singh SJ, Halpin DMG, Salvi S, Kirenga BJ, Mortimer K. Comment Exercise and pulmonary rehabilitation for people with chronic lung disease in LMICs : challenges and opportunities. *Lancet Respir* [Internet]. 2019;7(12):1002–4.

9. Nici L, Donner C, Wouters E, Zuwallack R, Ambrosino N, Bourbeau J, et al. American Thoracic Society Documents American Thoracic Society / European Respiratory Society Statement on Pulmonary Rehabilitation. *Am J Respir Crit Care Med*. 2006;173(May):1390-413.

10. Mccarthy B, Casey D, Devane D, Murphy K, Murphy E, Lacasse Y, et al. Pulmonary rehabilitation for chronic obstructive pulmonary disease (Review). *Cochrane Database Syst Rev*. 2015;2015(2).

11. Jones AW, Taylor A, Gowler H, Kelly NO, Ghosh S, Bridle C. Systematic review of interventions to improve patient uptake and completion of pulmonary rehabilitation in COPD. *ERJ Open Res*. 2017;3.

12. Chan AW, Tetzlaff JM, Altman DG, Laupacis A, Gøtzsche PC, Krleža-Jerić K, et al. Spirit 2013 statement: Defining standard protocol items for clinical trials. *Chinese J Evidence-Based Med*. 2013;13(12):1501–7.

13. Bolton CE, Bevan-Smith EF, Blakey JD, et al. British Thoracic Society guideline on pulmonary rehabilitation in adults: accredited by NICE. *Thorax*. 2013;68:ii1-ii30.

14. Ries AL, Bauldoff GS, Casaburi R, Mahler DA, Rochester CL, Herrerias C. Pulmonary Rehabilitation; Joint ACCP/AACVPR Evidence-Based Clinical Practice Guidelines. *Chest*. 2007;131(5):4S-42S.

15. Blackstock FC, Evans RA. Rehabilitation in lung diseases: 'Education' component of pulmonary rehabilitation. *Respirology*. 2019;24(9):863–70

16. Spencer LM, McKeough ZJ. Maintaining the benefits following pulmonary rehabilitation: Achievable or not? *Respirology*. 2019;24(9):909–15.

17. Armstrong M, Vogiatzis I. Personalized exercise training in chronic lung diseases. *Respirology*. 2019;24(9):854–62.

18. Revill SM, Morgan MDL, Singh SJ, Williams J, Hardman AE. The endurance shuttle walk : a new field test for the assessment of endurance capacity in chronic obstructive pulmonary disease. *Thorax*. 1999;54(2):213–22

19. Yasuda H, Yamaya M, Nakayama K, Ebihara S, Sasaki T, Okinaga S, et al. Increased

For peer review only - http://bmjopen.bmj.com/site/about/guidelines.xhtml

Arterial Carboxyhemoglobin Concentrations in Chronic Obstructive Pulmonary Disease. *Am J Respir Crit Care Med*. 2005;171:1246–51.

20. Durnin JVGA, Womersley J. Body fat assessed from total body density and its estimation from skinfold thickness: measurements on 481 men and women aged from 16 to 72 Years. *Br J Nutr*. 1974;32:77–96.
21. Doña E, Olveira C, Palenque FJ, Porras N, Dorado A, Martín-valero R, et al. Body Composition Measurement in Bronchiectasis: Comparison between Bioelectrical Impedance Analysis, Skinfold Thickness Measurement, and Dual-Energy X-ray Absorptiometry before and after Pulmonary Rehabilitation. *J Acad Nutr Diet.* 2018;118(8):1464–73.
22. Lanerolle MD, Lanerolle P, Atukorala S, Silva A De. Enhancing the accuracy of bio impedance analysis in assessing total body water. *Int J Body Compos* Res. 2012;10(1):29–34.
23. Bestall JC, Paul EA, Garrod R, Garnham R, Jones PW, Wedzicha JA. Usefulness of the Medical Research Council (MRC) dyspnoea scale as a measure of disability in patients with chronic obstructive pulmonary disease. *Thorax*. 1999;54:581–6.
24. Herdman M, Gudex C, Lloyd A, Janssen M, Kind P, Parkin D, et al. Development and preliminary testing of the new five-level version of EQ-5D (EQ-5D5L). *Quality of Life Research*. 2011;20(10):1727-736.
25. Van der Molen T, Willemse BW, Schokker S, et al. Development, validity and responsiveness of the Clinical COPD Questionnaire. *Health Qual Life Outcomes.* 2003;1:13.
26. Papaioannou M, Pitsiou G, Manika K, Kontou P, Zarogoulidis P, Sichletidis L, Kioumis IP. COPD assessment test: A simple tool to evaluate disease severity and response to treatment. *J of Chronic Obstructive Pulmonary Disease*. 2014;11:489- 95.
27. Reilly MC, Zbrozek IAS, Dukes EM. The Validity and Reproducibility of a Work Productivity and Activity Impairment Instrument. *Pharmacoeconomics*. 1993;4(5):353–65.
28. Zigmond AS, Snaith RP. The hospital anxiety and depression scale. *Acta Psychiatrica Scandinavica.* 1983;67(6):361-70.
29. Buysse DJ, Reynolds CF, Monk TH, et al. The Pittsburgh Sleep Quality Index: a new instrument for psychiatric practice and research. Psychiatry Res. 1989;28:193–213
30. Weisman IM, Marciniuk D, Martinez FJ, Sciurba F, Sue D, Myers J, et al. American Thoracic Society / American College of Chest Physicians ATS / ACCP Statement on Cardiopulmonary Exercise Testing. *Am J Respir Crit Care Med*. 2003;167:211–77.

For peer review only - http://bmjopen.bmj.com/site/about/guidelines.xhtml

31. Singh SJ, Morgan MD, Scott S, Walters D, Hardman AE. Development of a shuttle walking test of disability in patients with chronic airways obstruction. *Thorax*. 1992;47(12):1019-24.

32. Mahler DA, Horowitz MB. Perception of breathlessness during exercise in patients with respiratory disease. *Med Sci Sports Exerc*. 1994;26:1078–1081.

33. Borg GA. Psychophysical bases of perceived exertion. *Med Sci Sports Exerc*. 1982;14:377–381

34. Jones SE, Kon SSC, Canavan JL, Patel MS, Clark AL, Nolan CM, et al. The five-repetition sit-to-stand test as a functional outcome measure in COPD. *Thorax*. 2013;68:1015–20.

35. Booth M: Assessment of physical activity: An International perspective. *Research Quarterly for Exercise and Sport*. 2000, 71 (2): 114-120.

36. Bassett DR, John D. Use of pedometers and accelerometers in clinical populations: validity and reliability issues. *Phys Ther Rev*. 2010;15:135–42.

37. Choi L, Liu Z, Matthews CE, Buchowski MS. Validation of accelerometer wear and nonwear time classification algorithm. *Med Sci Sports Exerc*. 2011;43:357–64.

38. Demeyer H, Burtin C, Van Remoortel H, Hornikx M, Langer D, Decramer M, et al. Standardizing the analysis of physical activity in patients with COPD following a pulmonary rehabilitation program. *Chest* 2014 Aug;146(2):318-327

39. Troiano RP, Berrigan D, Dodd KW, Mâsse LC, Tilert T, McDowell M. Physical activity in the United States measured by accelerometer. *Med Sci Sports Exerc* 2008 Jan;40(1):181-188.

40. Lucas AO, Gilles HM. Approaches to economic evaluation. *Short Textbook of Public Health Medicine for the Tropics.* 2002. CRC Press.

41. Harris PA, Taylor R, Minor BL, Elliott V, Fernandez M, Neal LO, et al. The REDCap consortium: Building an international community of software platform partners. *J Biomed Inform*. 2019;95.

42. Braun V, Clarke V. Using thematic analysis in psychology.*Qual Res Psychol*. 2006;3:77–101

43. Maguire M, Delahunt B. Doing a Thematic Analysis : A Practical, Step-by-Step Guide for Learning and Teaching Scholars. *AISHE-J*. 2017;8(3):3351–14.

For peer review only - http://bmjopen.bmj.com/site/about/guidelines.xhtml

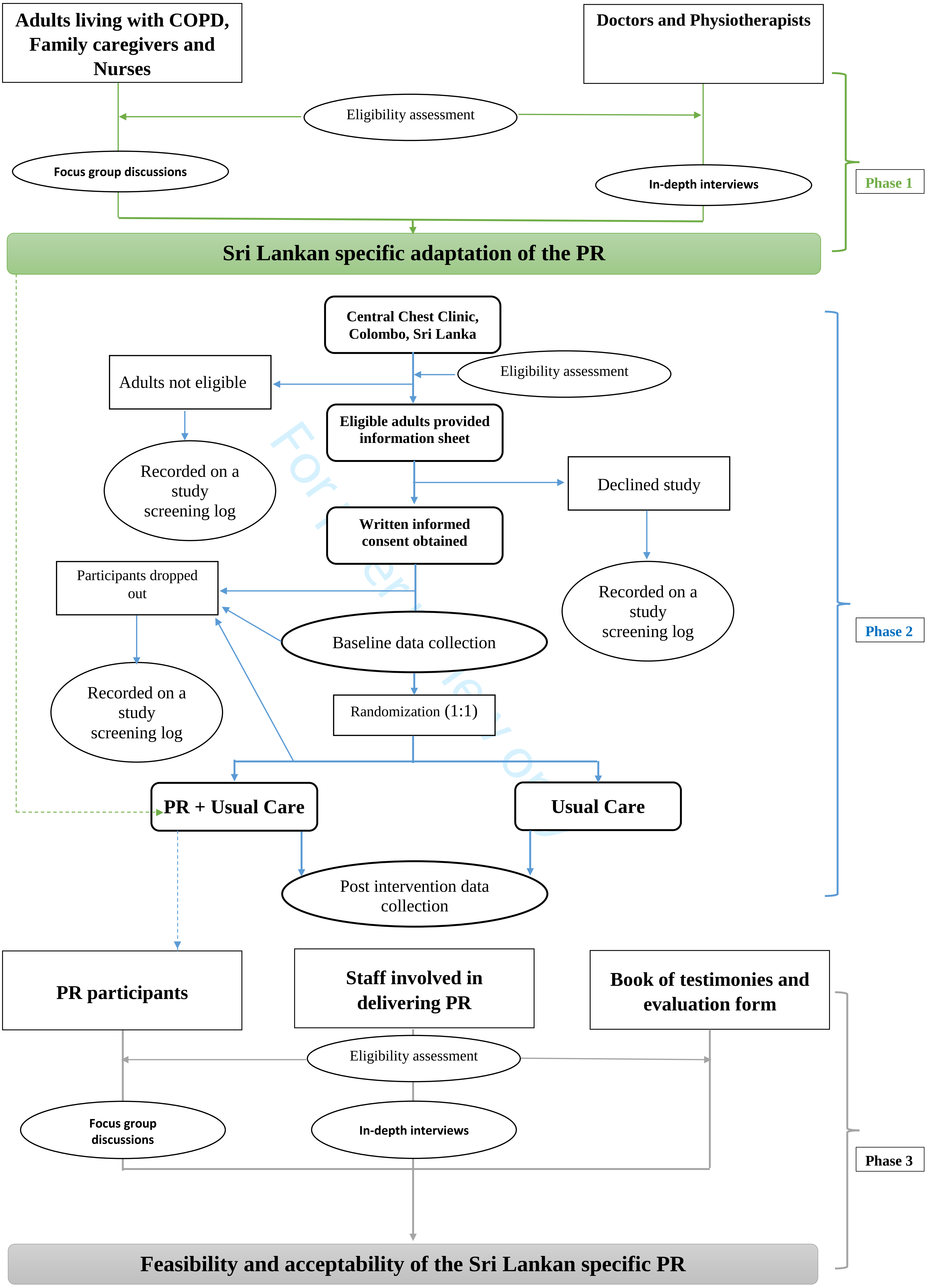
Adults living with COPD, Family caregivers and Nurses
Doctors and Physiotherapists
Eligibility assessment
Focus group discussions
In-depth interviews
Phase 1
Sri Lankan specific adaptation of the PR
Central Chest Clinic, Colombo, Sri Lanka
Eligibility assessment
Adults not eligible
Recorded on a study screening log
Eligible adults provided information sheet
Declined study
Written informed consent obtained
Recorded on a study screening log
Participants dropped out
Baseline data collection
Recorded on a study screening log
Randomization (1:1)
PR + Usual Care
Usual Care
Post intervention data collection
Phase 2
PR participants
Staff involved in delivering PR
Book of testimonies and evaluation form
Eligibility assessment
Focus group discussions
In-depth interviews
Phase 3
Feasibility and acceptability of the Sri Lankan specific PR

Appendices

Information sheet

Cultural adaptation of Pulmonary Rehabilitation and subsequent testing in a Randomised Controlled Feasibility Trial for Adults with Chronic Obstructive Pulmonary Disease in Sri Lanka

I am Prof. S, W. Wimalasekera, of Department of Physiology, Faculty of Medical Sciences, University of Sri Jayewardenepura. I and my team of health care personnel are, conducting a research on "Testing the Feasibility and Acceptability of a Culturally Appropriate Hospital-Based Pulmonary Rehabilitation (PR) Programme for Adults with Chronic Obstructive Pulmonary Disease in Sri Lanka". I invite you to participate in the study. Please take time and read the following information carefully before giving your consent. You are free to ask questions to clarify facts which are not clear or ask for more information if you need.

Purpose of the study

The purpose of this study is to devise an appropriate PR programme and then determine the feasibility and acceptability of this programme for adults living with COPD in Sri Lanka and assess the potential for a future trial of its effectiveness.

When you participate you will be required to do the following

You will be requested to participate several components of the study.

You will participate in a group discussion conducted in a quiet room in the clinic (conference hall) with very minimal disturbances. At the beginning of the discussion you will answer some questions regards your personal information, (age, gender, history of the disease condition). Then the interviewer will ask some questions regarding your thoughts and ideas about pulmonary rehabilitation. You and your group members can freely comment on the questions or can add your ideas to the discussion. The interview takes around forty-five minutes of time.

You will also participate in a feasibility trial which you will be allocated to one of the two groups. One group will receive usual care and PR and the other group will receive only usual care which consists of pharmacological treatment and brief information about disease condition, medication and inhaler techniques. PR is typically a 6-week rolling programme and consisting of 12 sessions in total, delivered twice a week. Sessions usually last approximately two hours, with one hour for exercise training and one hour for education. Some routine and specific test and measurements will be taken before and after the intervention will describe any changes in the health of the adults living with COPD.

If you are allocated to the group who receive the PR, you will be asked to share your experiences regarding the PR and subjected modification and opinions on pulmonary rehabilitation.

In order to maintain accuracy, and to keep records, your discussion will be recorded by the research team members with your permission. Your participation in the project will remain confidential and the provision of your name and address is necessary for us to maintain records. Any personal information gathered during the study will not be disclosed to a third party. Your responses to the questions and will

For peer review only

only be used for the purpose of this project. You can be assured that you will remain anonymous if you take part in the project. Your participation in this project is entirely voluntary. You are not obliged to take part. If you do not wish to take part, you can withdraw without giving a reason and your medical care will not be affected, further you will not be contacted again. Similarly, if you do agree to participate, you are free to withdraw at any time during the project.

What are the possible benefits of taking part?

By participating in the study, you will contribute to develop a PR programme which helps to improve the quality of life of the adults living with COPD. As well as you will contribute to determine the feasibility and acceptability of this programme within the health care system in Sri Lanka.

What are the possible disadvantages and risks of taking part?

You will be undertaking some physical tests as part of the research. Therefore, there may be a very small risk of falls, breathlessness, changes in blood pressure and changes in heart rate. However, these risks are very rare, and trained staff and emergency equipment will be available to deal with any serious events. Participants who experience any such event will be directed to the appropriate hospital and all the necessary care will be ensured and followed-up until the participant has resolved or stabilized.

What will happen if you don’t carry on with the study?

If you withdraw from the study all the information and data collected from you, to date, will be destroyed and your name would be removed from all the research study files. There will not be any loss or impact on your routine medical care which you are entitled to.

What will happen to the results of the research study?

The results of all the participants of the study will be used for scientific research. All data will be available only to the researchers and information provided will be strictly confidential. Your participation in the study is highly appreciated. Privacy and confidentiality of data gathered is completely secure and will be used only for research purposes. If you have any questions or any concerns with regards to the study and need further information you can call the telephone number listed below. Further you can send any complaint to the above postal address or email address.

Yours faithfully

Prof. S.W. Wimalasekera

Department of Physiology, Faculty of Medical Sciences, University of Sri Jayewardenepura

T.P No. 0777487203 e-mail : savithriww@yahoo.com

Ethics Review Committee – University of Sri Jayewardenepura - +94 11 2758000 Ext 4075e

For peer review only

Consent Form: **Randomised Controlled Feasibility Trial**

Cultural adaptation of Pulmonary Rehabilitation and subsequent testing in a Randomised Controlled Feasibility Trial for Adults with Chronic Obstructive Pulmonary Disease in Sri Lanka

1.Have you read the information sheet? (please keep a copy for yourself) YES/NO

2.Have you had an opportunity to discuss this and ask any questions? YES/NO

3. Have you had satisfactory answers to all your questions? YES/NO

4. Have you received enough information about the study? YES/NO

5. Who explained the study to you---

6. Do you understand that you are free to withdraw from the study at any time,

without having to give a reason YES/NO

7. All personal details will be treated as STRICTLY CONFIDENTIAL.

Do you give your permission for these individuals to have access to the records? YES/NO

8. Have you had sufficient time to come to your decision? YES/NO

9. Do you agree to take part in this study? YES/NO

Participant's signature --------------------------------------- Date------------------------

By the Witness

I have been present while the procedure was explained to the participant and I have witness his/ her willingness to take part in the study.

Signature of witness--- Date-------------------------------

(The witness should be a person not connected to the study)

Name --

Contact details---

For peer review only

BMJ Open

Protocol for the Cultural Adaptation of Pulmonary Rehabilitation and Subsequent Testing in a Randomised Controlled Feasibility Trial for Adults with Chronic Obstructive Pulmonary Disease in Sri Lanka

Journal:	*BMJ Open*
Manuscript ID	bmjopen-2020-041677.R2
Article Type:	Protocol
Date Submitted by the Author:	05-Nov-2020
Complete List of Authors:	Jayamaha, Akila; KIU, Health Sciences<br>Perera, Chamilya; KIU, Health Sciences<br>Orme, Mark; University Hospitals of Leicester NHS Trust, Centre for Exercise and Rehabilitation Science, NIHR Leicester Biomedical Research Centre-Respiratory; University of Leicester, Department of Respiratory Sciences<br>Jones , Amy; University Hospitals of Leicester NHS Trust, Centre for Exercise and Rehabilitation Science, NIHR Leicester Biomedical Research Centre-Respiratory; University of Leicester, Department of Respiratory Sciences<br>Wijayasiri, Upendra; Colombo South Teaching Hospital, Sports Medicine Unit<br>Amarasekara, Thamara; University of Sri Jayewardenepura, Faculty of Allied Health Sciences<br>Karunatillake, Ravini ; National Hospital of Sri Lanka, Central Chest Clinic<br>Fernando, Amitha; National Hospital of Sri Lanka, Central Chest Clinic<br>Seneviratne, Anthony ; Primary Care Respiratory Group<br>Barton, Andy; University of Plymouth, Faculty of Health<br>Jones, Rupert; University of Plymouth, Faculty of Health<br>Yusuf, Zainab; Centre for Exercise and Rehabilitation Science, NIHR Leicester Biomedical Research Centre-Respiratory, University Hospitals of Leicester NHS Trust; University of Leicester, Department of Respiratory Sciences<br>Miah, Ruhme ; University of Leicester, Department of Respiratory Sciences; University Hospitals of Leicester NHS Trust, Centre for Exercise and Rehabilitation Science, NIHR Leicester Biomedical Research Centre-Respiratory<br>Malcolm, Dominic; Loughborough University, School of Sport, Exercise and Health Sciences<br>Matheson, Jesse; University of Sheffield, Department of Economics<br>Free, Robert; University of Leicester, Department of Respiratory Sciences,<br>Manise, Adrian ; University of Leicester, Department of Respiratory Sciences<br>Steiner, michael ; University of Leicester, Department of Respiratory Sciences; University Hospitals of Leicester NHS Trust, Centre for Exercise and Rehabilitation Science, NIHR Leicester Biomedical Research Centre-Respiratory

	Wimalasekera, Savithri; University of Sri Jayewardenepura, Faculty of Medical Sciences<br>Singh, Sally; University of Leicester, Department of Respiratory Sciences; University Hospitals of Leicester NHS Trust, Centre for Exercise and Rehabilitation Science, NIHR Leicester Biomedical Research Centre-Respiratory
**Primary Subject Heading**:	Respiratory medicine
Secondary Subject Heading:	Rehabilitation medicine
Keywords:	REHABILITATION MEDICINE, Chronic airways disease < THORACIC MEDICINE, Emphysema < THORACIC MEDICINE, Protocols & guidelines < HEALTH SERVICES ADMINISTRATION & MANAGEMENT

I, the Submitting Author has the right to grant and does grant on behalf of all authors of the Work (as defined in the below author licence), an exclusive licence and/or a non-exclusive licence for contributions from authors who are: i) UK Crown employees; ii) where BMJ has agreed a CC-BY licence shall apply, and/or iii) in accordance with the terms applicable for US Federal Government officers or employees acting as part of their official duties; on a worldwide, perpetual, irrevocable, royalty-free basis to BMJ Publishing Group Ltd ("BMJ") its licensees and where the relevant Journal is co-owned by BMJ to the co-owners of the Journal, to publish the Work in this journal and any other BMJ products and to exploit all rights, as set out in our licence.

The Submitting Author accepts and understands that any supply made under these terms is made by BMJ to the Submitting Author unless you are acting as an employee on behalf of your employer or a postgraduate student of an affiliated institution which is paying any applicable article publishing charge ("APC") for Open Access articles. Where the Submitting Author wishes to make the Work available on an Open Access basis (and intends to pay the relevant APC), the terms of reuse of such Open Access shall be governed by a Creative Commons licence – details of these licences and which Creative Commons licence will apply to this Work are set out in our licence referred to above.

Other than as permitted in any relevant BMJ Author's Self Archiving Policies, I confirm this Work has not been accepted for publication elsewhere, is not being considered for publication elsewhere and does not duplicate material already published. I confirm all authors consent to publication of this Work and authorise the granting of this licence.

For peer review only

Protocol for the Cultural Adaptation of Pulmonary Rehabilitation and Subsequent Testing in a Randomised Controlled Feasibility Trial for Adults with Chronic Obstructive Pulmonary Disease in Sri Lanka

Akila R Jayamaha [1], Chamilya H Perera [1], Mark W Orme [2,3], Amy V Jones [2,3], Upendra K D C Wijayasiri [4], Thamara D Amarasekara [5], Ravini de S Karunatillake [6], Amitha C Fernando [6], Seneviratne A L P De S.[7], Andy Barton [8], Rupert Jones [8], Zainab K Yusuf [2,3], Ruhme B Miah [2,3], Dominic Malcolm [9], Jesse A Matheson [10], Robert C Free [3], Adrian Manise [3], Michael C Steiner [2,3] Savithri W Wimalasekera [11], Sally J Singh [2,3]

Correspondence to Akila Randika Jayamaha/1226arj@gmail.com/akila@kiu.ac.lk

Abstract

Introduction International guidelines recommend pulmonary rehabilitation (PR) should be offered to adults living with COPD, but PR availability is limited in Sri Lanka. Culturally appropriate PR needs to be designed and implemented in Sri Lanka. The study aims to adapt PR to the Sri Lankan context and determine the feasibility of conducting a future trial of the adapted PR in Sri Lanka.

Methods and analysis Eligible participants will be identified and will be invited to take part in the randomised controlled feasibility trial, which will be conducted in Central Chest Clinic, Colombo, Sri Lanka. A total of 50 participants will be recruited (anticipated from April 2021) to the trial and randomised (1:1) into one of two groups; control group receiving usual care or the intervention group receiving adapted PR. The trial intervention is a Sri Lankan specific PR programme, which will consist of 12 sessions of exercise and health education, delivered over 6 weeks. Focus groups with adults living with COPD, caregivers and nurses and in-depth interviews with doctors and physiotherapist will be conducted to inform the Sri Lankan specific PR adaptations. After completion of PR, routine measures in both groups will be assessed by a blinded assessor. The primary outcome measure is feasibility, including assessing eligibility, uptake and completion. Qualitative evaluation of the trial using focus groups with participants and in-depth interviews with PR deliverers will be conducted to further determine feasibility and acceptability of PR, as well as the ability to run a larger future trial.

Ethics and dissemination Ethical approval was obtained from the ethics review committee of Faculty of Medical Sciences, University of Sri Jayewardenepura, Sri Lanka and University of

For peer review only - http://bmjopen.bmj.com/site/about/guidelines.xhtml

Leicester, UK. The results of the trial will be disseminated through patient and public involvement events, local and international conference proceedings, and peer-reviewed journals.

Trial registration:ISRCTN13367735

Keywords: COPD, Pulmonary Rehabilitation, Exercise, Education, Sri Lanka

Strengths and limitations

- This study is the first examining the feasibility and acceptability of a culturally appropriate pulmonary rehabilitation programme for adults with COPD in Sri Lanka.
- Taking a mixed-method approach, this study will provide a rich insight into delivering a trial of pulmonary rehabilitation in the Sri Lankan context.
- This study is a single center feasibility trial. As such, whilst findings will be an important first step in understanding the potential role of pulmonary rehabilitation in Sri Lanka and low- and middle-income countries more broadly, findings may not be generalizable to other regions of the world.

For peer review only

INTRODUCTION

Chronic obstructive pulmonary disease (COPD) is a progressive disease characterised by airflow obstruction and breathlessness. COPD is a major cause of morbidity and mortality throughout the world, corresponding to 6% of all deaths worldwide.[1] Further, more than 90% of COPD deaths occur in low and middle-income countries.[2] The most recent estimate of COPD prevalence in South Asia was 6.3% [3] with a prevalence in Sri Lanka of 10.5%,[4] similar to the estimated global COPD prevalence of 11.7%.[5] COPD is a significant burden to both patients and healthcare services.[6]

Chronic cough with sputum, breathlessness, physical inactivity and exercise intolerance resulting from dyspnoea or fatigue are common consequences of COPD.[1] Symptoms of COPD progressively worsen and people can become breathless, even at rest. Daily activities often become difficult as the condition worsens, impacting their quality of life.[7] The impact of COPD to the individual and to society makes the need for interventions to reverse the associated disability of paramount importance.

International guidelines recommend that pulmonary rehabilitation (PR) should be routinely offered to patients with chronic respiratory disease who have persistent symptoms, limited activity, and/or are unable to adjust to illness.[8,9] It is a low cost, high impact intervention that improves the quality of life, reduces suffering, reduces mortality and reduces economic loss, relieves dyspnoea and fatigue, improves exercise capacity, improves psychological and emotional function, and enhances an individual's self-management of their condition.[1] Having realised the benefits of PR in COPD, Western countries have incorporated this as an important structural component of healthcare delivery services.[10] Implementation of PR based as practiced in Western countries, in Sri Lanka requires adaptation to the local health service, population and culture. Despite its effectiveness, there is a significant need to understand the feasibility of conducting PR in low resource Sri Lankan health care setting and the acceptability of PR among Sri Lankan adults living with COPD and healthcare staff involved in its delivery. Uptake and completion of PR even in Western countries is an on-going challenge.[11] The need to maximize appeal of PR to patients and referrers is a global issue and one that is likely to be specific to a given location and population. There remains an unmet need for PR in Sri Lanka. To be

For peer review only

For peer review only - http://bmjopen.bmj.com/site/about/guidelines.xhtml

successful, PR must be not only evidence-based, but also designed and implemented in a manner sensitive to the context in which it is being delivered, such as culture and geography. Therefore, the aim of this study is to devise an appropriate PR programme and then determine the feasibility and acceptability of this programme for adults living with COPD in Sri Lanka and assess the potential for a future trial of its effectiveness.

The objectives of the study are to:

1. Explore the needs and perceptions of adults living with COPD, their care-givers /family members and health care professionals to inform the adaptations required for a PR programme suitable for the Sri Lankan context.
2. Determine the feasibility of conducting adapted hospital-based PR for people living with COPD.
3. Assess the acceptability of the PR among Sri Lankan adults living with COPD and healthcare staff involved in its delivery.
4. Describe any changes in health of the adults living with COPD following completion of PR.
5. Assess the feasibility of a future trial and estimate the required sample size.

METHODS AND ANALYSIS

Study design and registration

The proposed study will be conducted in three phases. Phase 1: A qualitative study will be conducted to inform the adaptations required to make PR specific to the Sri Lankan context. Phase 2 is a single blind randomised control feasibility trial. Adults living with COPD will be randomised (1:1) into adapted PR or usual care. Phase 3: Qualitative evaluation of the trial to determine the feasibility and acceptability of PR deliverers and participants. The trial will be conducted, analysed and reported according to the Standard Protocol Items: Recommendations for Interventional Trials (SPIRIT) statement [12] and trial has been registered on the ISRCTN website. Study was commenced upon ethical approval on 24/07/2020 for Phase 1 of the study. The design of the study and flow of participant enrolment is presented in figure 1.

For peer review only - http://bmjopen.bmj.com/site/about/guidelines.xhtml

Study setting

The study will be carried out at the Central Chest Clinic, Colombo, Sri Lanka which is a leading health care facility that provides treatment for adults with respiratory disease. A room allowing confidential discussions will be used for conducting focus group discussions among adults living with COPD, their caregivers /family members, and nurses. Semi structured interviews will be conducted among doctors and physiotherapists in a given quite room at the Central Chest Clinic as convenient to them and without interfering with their routine work. The venue for PR will be a large room at the Central Chest Clinic.

Participants

Key informants and suitable participants for phase 1 of the study will be identified by the researchers with the help of health care professionals involved in the treatment of COPD at Central Chest Clinic. Suitable participants will be purposively selected and informed verbally about the study by the researchers. After receiving a study information sheet, potential participants will be contacted to arrange an appointment, if they wish to take part. An opportunity to ask questions will be provided. If willing to take part in the study, they will be asked to provide written informed consent. Multi-religious and multi-ethnic Sri Lankan cultural diversity and gender will be considered when recruiting to the study. Adults living with COPD aged ≥18 years and Medical Research Council (MRC) dyspnoea score grade 2 or higher, Family member aged ≥18 years and looks after a patient with COPD and Health care professionals who have more than 1-year experience of managing patients with COPD and working in the government health care system of the country will be eligible to participate in the phase 1 of the study. Participants who provide written informed consent will be enrolled in the study.

After the adaptation of PR in phase 1, suitable participants for the Phase 2 randomised controlled feasibility trial will be identified by the Medical Officers in the Chest Clinic and will be invited to take part in an eligibility assessment. This assessment will determine eligibility for entry into the study. People eligible for inclusion in the trial will be: aged ≥18 years, will have a clinically confirmed diagnosis of COPD by a physician, confirmed COPD with spirometry based on GOLD criteria with $FEV_1/FVC<0.7$, and $FEV_1<80\%$ predicted, ≥1 exacerbation required a hospitalisation in the year preceding study, mMRC grade ≥2 and willing to provide informed consent. Adults with comorbidities such as severe or unstable cardiovascular, other internal

For peer review only - http://bmjopen.bmj.com/site/about/guidelines.xhtml

diseases and locomotor difficulties that preclude the exercise or malignant disease or other serious illness which will interfere with participation in the PR, will be excluded from the study. Individuals not eligible for the study will be recorded on a study screening log.

[insert figure 1 here]

Procedure

Eligible participants will be informed verbally about the study by the Medical Officers of the Central Chest Clinic. After receiving a study information sheet, potential participants will be contacted to arrange an appointment, if they wish to take part. An opportunity to ask questions will be provided. If willing to take part in the study, they will be asked to provide written informed consent. Information regarding the interest of participation in the study will be taken as field notes. Baseline outcome measures will be assessed. After baseline data collection, participants will be randomised (1:1) into two groups. One group will receive usual care and PR and the other group will receive only usual care which consists of pharmacological treatment and brief information about disease condition, medication and inhaler techniques. Experiences of the participants and PR deliverers regarding the acceptability and feasibility of PR will be explored in interviews and focus groups during Phase 3 of the study. Participants who did not complete the PR will be asked to take part in a drop-out interview and information provided freely by the participants will be collected as field notes.

Trial interventions

Intervention: Adapted pulmonary rehabilitation

Sri-Lankan specific PR will comprise the core elements of an evidence based rehabilitation, a programme of exercises [13, 14] and health education.[13, 14, 15] The detail of delivery and adaptations of PR will be informed by findings of the Phase 1 of the study. PR is typically a 6-week rolling programme that consists of 12 sessions in total.[13, 14] Sessions usually last approximately two hours, with one hour for exercise training and one hour for education. All the education sessions and the instructions during PR will be provided in Sinhala language as convenient to the participants. The duration of the PR trial and frequency and duration of sessions will be

For peer review only - http://bmjopen.bmj.com/site/about/guidelines.xhtml

discussed during Phase 1. All staff delivering PR will be trained and assisted by a nursing officer, a clinical nutritionist, an exercise physician and a physiotherapist. The venue for PR will be at the Central Chest Clinic, Colombo, Sri Lanka. The Central Chest Clinic will have a maximum capacity of 10 patients per PR class. The equipment required will be simple and include chairs, weights, and simple exercise equipment based on local availability, suitability and informed by qualitative work.[16]

In keeping with evidence-based guidelines [13, 14], discussions in Phase 1 will include the core exercise component of PR, notably resistance training (e.g. upper and lower limbs) and aerobic training (walking, sit-to-stand, step-ups) using minimal equipment and education. Each participant's exercise regimen will be individually prescribed and progressed [17] with walking speed will be prescribed using the ISWT.[18] The process in which this is achieved by patients will be informed by Phase 1. The education component will be delivered by an interdisciplinary team and will be informed by qualitative findings from Phase 1 with discussions facilitated by usual education topics as described in guidelines.[13, 14]

Usual care

Usual care will consist of pharmacological treatment and optimization, prescribed to patients after consultation with Medical Officers at the Central Chest Clinic. Brief information about disease condition, medication and inhaler techniques will be provided by Medical Officers and Nurses. Pulmonary rehabilitation or any form of exercise recommendation is not part of usual care.

Outcomes

Primary outcome

The primary outcome of the trial will be the feasibility and the acceptability of the PR intervention.

Feasibility

For peer review only - http://bmjopen.bmj.com/site/about/guidelines.xhtml

188 Measures to assess feasibility are provided in table 1 and include the comprehensive assessment
189 of the feasibility of patient recruitment and the intervention delivery.

**Table 1: Primary outcome measures- Feasibility and operational experience assessment**	
**Feasibility of patient recruitment**	**Data sources**
Feasibility of screening and recruiting participants	Interviews with the healthcare professionals, screening log
Suitability of the inclusion criteria	Interviews with the healthcare professionals, screening log
Number of eligible patients, number of patients screened, number of patients invited to take part, actual number of participants who consent to take part	Screening log
Number of patients who refuse, drop out and the reasons for refuse and drop out	Interviews with the patients, screening log
**Operational experience of intervention delivery**	
Service provider and multi-disciplinary teams' willingness and ability to deliver the PR	Interviews with healthcare professionals
The practicality of delivering the intervention in the proposed setting	Interviews with healthcare professionals and focus groups with participants
The time needed to collect the data<br>Baseline visit- Time taken for each measure (each individual questionnaire and physical measure)<br>Follow-up visit- Time taken for each measure (each individual questionnaire and physical measure)	Interviews with the healthcare professionals, Rehabilitation records
Data completeness and accuracy	Interviews with the healthcare professionals, Rehabilitation records, RedCap
Adherence to home exercise	Interviews with the patients and self-report exercise diary
The training and resources needed to deliver the intervention (ensuring readiness for a future much larger multi-center trial)	Interviews with the healthcare professionals and focus groups with participants, Rehabilitation records
Description of unintended events	Adverse events log, REDCap

190 ## Acceptability

191 The acceptability of the PR intervention among adults living with COPD and healthcare staff
192 involved in its delivery will be assessed. Participants' experience of the PR, including any
193 perceived benefits, challenges and changes they would make to the programme, will be explored
194 in qualitative interviews and focus groups after their discharge assessment or withdrawal. The
195 experience of healthcare professionals regarding the PR intervention, such as their confidence in
196 delivering the programme, the components of PR, structure of PR, the patient adherence to the

For peer review only - http://bmjopen.bmj.com/site/about/guidelines.xhtml

197 PR exercises and how their perceptions changed over the course of the trial, insights into barriers and facilitators to referral, uptake and completion of PR ((i) attending at least 10 out of 12 designated PR sessions and (ii) attending the follow-up evaluation) will be explored in qualitative interviews at the end of the trial.

201 Secondary outcomes

202 The secondary outcomes of this study are provided in table 2. Comparison of secondary outcome measures of baseline and post intervention, will describe any changes in the health of the adults living with COPD following completion of PR

Table 2: Secondary outcome measures

**Outcome measures**	**Baseline**	**Post-intervention**
Socio-demographics	x	
Lung health (spirometry data, smoking status, number of COPD exacerbations in the last year)	x	
Comorbidities	x	
Treatments	x	
Nutritional status (Body Mass Index, mid upper arm circumference, skinfold thickness, self-report seven-day diet diary)	x	x
Disease burden (MRC dyspnea grade, CAT, CCQ)	x	x
Economic impact of disease (WPAI)	x	x
Quality of life (EQ-5D-5L)	x	x
Pittsburgh Sleep Quality Index (PSQI)	x	x
Psychological wellbeing (Hospital Anxiety and Depression scale)	x	x
Physical function (5x sit-to-stand test)	x	x
Exercise capacity (ISWT, ESWT)	x	x
International Physical Activity Questionnaire (IPAQ)	x	x
Accelerometer assessed physical activity (ActiGraph wGT3x-BT)	x	x

205 MRC - Medical Research Council, CAT- COPD Assessment Test, CCQ - Clinical COPD Questionnaire, WPAI – Work Productivity and Activity Impairment questionnaire, EQ-5D-5L - EuroQol Five Dimensions Five Levels, ISWT - Incremental Shuttle Walk Test, ESWT - Endurance Shuttle Walk Tests

208 Sample size and recruitment target

209 This study is a feasibility trial that aims to provide data for an accurate estimation of the required sample size for future trials. Therefore, a formal sample size calculation is not required. We aim to recruit and randomise 50 participants to the study (25 in each group).

For peer review only - http://bmjopen.bmj.com/site/about/guidelines.xhtml

Patient allocation, concealment, and blinding

Randomisation will occur through random permuted blocks to either the intervention group (PR+ usual care) or control group (usual care) in ratio 1:1 using Sealed envelope™.

The computer-generated patient allocation sequence will be monitored by an individual who is independent of the research team and will inform the research team of group allocations via telephone. Participants will be informed about their group allocation after providing informed consent and completing baseline assessments.

It will not be possible to blind patients to their group allocation due to the nature of PR. Research staff will be blinded to outcome measures. Participants will be advised not to reveal their group during the follow-up assessment. Any episodes of un-blinding will be documented and reported.

Data collection

Phase 1: Qualitative assessment for adaptation of PR

Focus groups with patient and family caregivers

Focus groups with adults living with COPD and separate focus groups with their family members/ caregivers will be conducted until data saturation. We anticipate conducting up to 5 focus groups with 6-8 participants in each. Data will be collected during patients' clinic visits. Focus group discussions will be audio-recorded, expected to last approximately 45-90 minutes, and will be conducted face-to-face by an interviewer and note-taker (observer). Focus groups will be transcribed verbatim, with identifiable information removed. Envisaged outcomes of the focus groups will include Sri Lankan specific adaptation of PR.

Interviews with healthcare professionals involving the treatment of COPD

Up to 15 in-depth interviews with healthcare professionals will be conducted until data saturation. Structured interviews will be conducted with healthcare professionals as convenient to them without interfering with their routine work. Interviews will be audio-recorded and will be conducted face-to-face by an interviewer. Interviews will be transcribed verbatim, with

For peer review only - http://bmjopen.bmj.com/site/about/guidelines.xhtml

identifiable information removed. Envisaged outcomes of the in-depth interviews will be a suitable design and content of PR acceptable respiratory health care deliverers in Sri Lanka.

Phase 2: Single blind randomised control feasibility trial

Data will be collected by trained researchers, following standard operating procedures during participants' clinic visits. Baseline and post intervention assessments will be carried out by the blinded Medical Officers consulting at the study setting.

Phase 3: Qualitative evaluation of the PR intervention

Focus groups with patients

Participants allocated to the intervention group will be invited to participate in focus group discussions at the end of their PR programme. Focus groups will give an insight on views, experiences, opinions and recommendations which will inform future PR programmes. We anticipate conducting up to 5 focus groups until data saturation. Each focus group discussion will be conducted with 6-8 participants in each.

Focus group discussions will be audio-recorded, expected to last approximately 45-90 minutes, and will be conducted by a trained moderator and a note-taker). Focus groups will be transcribed verbatim, with identifiable information removed. Consent will be obtained from participants prior to their involvement in focus groups.

Interviews with PR staff

Health care personnel involved in delivering PR will be invited to participate in in-depth interviews at the end of the study to discuss aspects of feasibility and acceptability, such as insights into barriers and facilitators to attendance, logistical barriers of running a PR programme and their perceptions, confidence of programme delivery and patients' experiences of the intervention. Details regarding previous experience on PR and prior training regarding PR will be assessed using brief questionnaire before commencing the in-depth interviews. We anticipate conducting up to 15 interviews, each expected to last approximately 15-60 minutes. Interviews

For peer review only - http://bmjopen.bmj.com/site/about/guidelines.xhtml

will be audio-recorded and will be conducted face-to-face by a trained interviewer. Interviews will be transcribed verbatim, with identifiable information removed.

Book of testimonies and evaluation form

Participants within PR will be asked to log their experience of PR as they progress through the programme. This will be in the form of a PR log book accessible to participants before, during and after sessions, as well as a dedicated evaluation form which will be provided as supplementary material. Staff involved in PR will also receive the same evaluation form at the end of the study.

Sample characteristics

Basic demographics including age, sex, religion, nationality, marital status, age of leaving full-time education, education level, ethnicity, employment status, monthly income, lung health, smoking status (packs per year), biomass fuel exposure, primary respiratory diagnosis, time since diagnosis in years, secondary respiratory diagnoses, family history of lung disease, comorbidities will be recorded at baseline.

Lung function

Spirometry (post bronchodilator Forced Expiratory Volume in the first second (FEV_1), post bronchodilator Forced Vital Capacity (FVC), FEV_1/FVC ratio, carboxyhemoglobin test [19], hospitalisations within the last 12 months, number of COPD exacerbations within the last 12 months, treatments will be collected as the baseline data prior to the randomisation of participants.

Nutritional status

Height (Holtain stadiometer), weight (calibrated weight scale) will be measured and body mass index will be calculated. Mid upper arm circumference of the participants will be measured using a measuring tape and skinfold thickness of seven sites (triceps, mid axillary, subscapular, chest, abdominal, supra iliac and thigh measurements) of the body will be measured using a validated caliper. Triplicated measurements will be taken by the same investigator and mean value will be

For peer review only - http://bmjopen.bmj.com/site/about/guidelines.xhtml

used for the calculation of body fat mass. Fat free mass will be calculated using Durnin and Womersley formula.[20, 21] Body composition analysis will be assessed using bioelectrical impedance (SFB7 Impedi Med dual frequency instrument, Impedi Med Limited, Australia) and software version 5.2.4.0.[22] Seven-day diet diary method will be used to assess the dietary history and calorie intake.

Disease burden

Breathlessness will be measured using MRC dyspnoea grade (5-items).[23] The health-related quality of life related to mobility, self-care, usual activities, pain/discomfort and anxiety-depression will be measured using EQ-5D-5L (25-items),[24] Clinical status of the airways, functional limitations and psychosocial dysfunction will be determined using CCQ (10 -items),[25] CAT (8-items) will be used to determine the severity of the COPD,[26] Modified brief pain inventory (3-items) will determine the commonly reported chest pain, WPAI (8-items) will be used to measure the effect of severity of COPD and general health on work productivity and regular activities [27] and HADS (14-items) will be used to measure the patient's emotional state and the presence or absence of clinically significant anxiety and depression.[28] Sleep quality of the participants will be measured using Pittsburgh Sleep Quality Index (PSQI) [29]. Disease burden will be measured as baseline data prior to the randomisation and post-intervention data.

Exercise capacity

Following a safety assessment for absolute and relative contraindications for field walking tests[30], maximal exercise capacity will be assessed using the incremental shuttle walking test (ISWT) [31] and endurance shuttle walking test (ESWT).[18] The ISWT and ESWT also include a pre-test and post-test measure of SpO_2, blood pressure, Borg rating of breathlessness [32], heart rate, Borg rate of perceived exertion.[33] The sit-to stand test will be completed for five continuous repetitions, to measure lower limb movement and strength.[34] All measures of exercise capacity will be measured at baseline prior to the randomisation and post-intervention.

Physical activity

Subjective health–related physical activity will be measured with the International Physical Activity Questionnaire (IPAQ) [35] at baseline prior to the randomisation and post-intervention.

For peer review only - http://bmjopen.bmj.com/site/about/guidelines.xhtml

Participants will be asked to wear an activity monitor (ActiGraph wGT3X-BT, ActiGraph, Pensacola, FL, USA), able to detect a range of PA intensities [36], on the right anterior hip during waking hours for seven days prior to attending PR (baseline) and for the seven consecutive days prior to their discharge assessment (follow-up); taking it off for water-based activities and sleep. The collection of physical activity data will coincide with a 7-day diet diary. Printed instructions will be provided to the participants regarding how to wear the monitor.

Full accelerometry methodology is provided in Table 3. Data will be processed as 60-s epoch files in ActiLife version 6.13.4 (ActiGraph, Pensacola, FL, USA). Non-wear will be defined as 60-minutes of consecutive zero counts with allowance for 2-minutes of interruptions.[37] Adherence to wearing the monitors will be assessed by examining the proportion of participants providing valid accelerometer data across a range of valid days (≥1-7 days) and minimum wear time (≥1-12 hours) thresholds. For reported group averages, only participants providing ≥4 valid days of ≥8 hours, for both time points will be included in the analyses.[38] Step count, time spent in different absolute intensity classifications of physical behaviours [39] and average movement intensity (activity counts per minute) will be reported. Time spent in physical activity matching participants' prescribed walking exercise intensity will be derived by aligning the average walking speed during the ESWT with the activity monitor counts per minute.

Table 3: Accelerometry data collection and processing parameters

Accelerometer Model	ActiGraph wGT3X-BT (version 6.13.4; firmware 1.9.2)
Serial number range	Twenty unique devices will be used ranging from MOS2E09190601 to MOS2E24190146 averaging six deployments per device (same serial used for baseline and follow-up wear periods)
Piezosensor orientation	Triaxial
Mode setup	Mode 29 (x, y, z, steps, lux)
Original sample rate	100 Hz (.gt3x file format)
Deployment method	Baseline:<br>Fitted by research team on Day 0 (Baseline PR Assessment)<br>Fitted by participant on Day 1<br>Follow-up:<br>Fitted by research team Day 0 ($11^{th}/12^{th}$ session)<br>Fitted by participant on Day 1
Location worn	Anterior hip adjacent to the mid-line of the thigh
Requested days of wear	7 days of free-living (Day 1-7; 10,080 epochs)

For peer review only - http://bmjopen.bmj.com/site/about/guidelines.xhtml

Initialization	Not deployed in delay mode in order to standardised capture of Day 0 (00:00) with stop time based on date of first PR class (baseline) and data of discharge assessment (follow-up)
Wear instructions	Wear continuously except for sleep and water based activities
Non-wear appropriation	≥60 min of consecutive 0s with allowance for 2 minutes of interruptions were deemed biologically implausible and coded as non-wear
Valid day criteria	≥8 hours of valid waking wear time
Valid file	≥4 valid days for each of the two time points
Missing data	Data modelling or imputation will not be performed
Epoch length	60 seconds
Intensity classification (absolute)	Uniaxial (x-axis) cut-points as follows: Sedentary time <100 cpm; Light intensity 100-2019 cpm; Moderate intensity 2020-5998cpm; Vigorous intensity ≥5999cpm (Moderate-to-vigorous intensity ≥2020 cpm)
Intensity classification (relative)	Uniaxial (x-axis) cut-points based on Endurance Shuttle Walk Test performance

Patient and Public Involvement

Adults living with COPD often tell us how having COPD impacts their lives and that they often find it challenging to know what they can do to better manage their condition. When they talk about their self-management strategies, it is clear that education and exercise support would be of value. Also, they revealed the necessity of a programme to support their condition and willingness to attend such a programme. Patients are generally positive about being able to access support but there is little available to them. The study was planned to fulfill the need of adults living with COPD. The delivery and adaptations of the trial intervention (Sri-Lankan specific PR) will also be informed by the adults living with COPD, their care-givers /family members and health care professionals. Priorities, experience, and preferences of the stakeholders will be utilized to design the PR. Health care professionals involved in the treatment of COPD at Central Chest Clinic will be involved in the recruitment as key informants and conduct of the study. The feasibility and acceptability of the PR intervention among adults living with COPD and healthcare staff involved in its delivery will be assessed in qualitative interviews at the end of the trial. The results of the trial will be disseminated through patient and public involvement events, local and international conference proceedings. As well as all the research

For peer review only - http://bmjopen.bmj.com/site/about/guidelines.xhtml

participants, stakeholders and individuals with COPD will be openly invited to take part in an event organized at the central chest clinic to reveal the study findings.

Costing

As part of this trial, the cost of creating a Sri-Lankan adapted PR programme will be calculated. This will include both single and recurrent costs. Single payments include the necessary costs to set up and run a programme, whilst recurrent cost refers to any item with a life expectancy of 1 year or less and typically includes disposable materials.[40] The fixed costs will be captured prior to the first participant enrolling into the programme and the recurrent costs will be collected at the mid-stage of recruitment. An average cost per participant will be calculated. Table 4 demonstrates the variables that will be used to calculate fixed and recurrent costs.

Table 4: The variables used to calculate fixed and recurrent costs (not an exhaustive list)

Fixed costs	Recurrent costs
Venue hire	Venue hire
Electrical equipment (laptop, printer, projector)	Staff time to conduct PR (assessment at baseline and discharge, conduct PR classes, telephone calls and data entry)
Equipment for PR (weights, treadmill, cycle ergometer, country-specific equipment, step-up box, chairs)	Disposable equipment (for blood glucose monitor, spirometer mouthpieces, nose-clips, glyceryl trinitrate spray)
Equipment for shuttle walking tests (cones, licences, stop watches, tape measure, electrical equipment to play audio)	Servicing costs (spirometer, PR equipment, specifically treadmills and cycle ergometers)
Equipment for PR assessment (height stadiometer, weight scales, sphygmomanometer, pulse oximeter, spirometer, calibration syringe, country-specific equipment)	Miscellaneous (Oxygen cylinders, questionnaire licences, stationery (paper))
Additional safety equipment (blood glucose monitor, Oxygen cylinder holder)	

For peer review only - http://bmjopen.bmj.com/site/about/guidelines.xhtml

Miscellaneous (filing cabinets, storage units, questionnaire translations, questionnaire licences, staff uniform)	

Data management

Data collected during the study will be entered into a database using Research Electronic Data Capture (REDCap), which is a web-based platform.[41] Access to the database will be via a secure password protected web-interface. The participants will be identified by a study-specific identification code. Data will be validated using real-time data entry validation and electronic checks lead by the Independent Data Monitoring Committee (IDMC), established at the University of Leicester, UK.

Quantitative data analysis

The data will be analyzed using IBM SPSS Statistics for Windows, Version 23.0. Data for baseline and follow-up time-points will be presented as descriptive statistics as appropriate. No inferential statistics will be performed due to the feasibility design of the trial.

Qualitative data analysis

Qualitative data will be analyzed using Thematic Analysis. This approach follows six distinct stages: familiarization with data; generating initial codes; searching for themes; reviewing themes; defining and naming themes and producing the report.[42] The responsible investigator will carry out initial coding and a sample of focus group transcripts will be coded by a second member of the team to improve consistency and to enhance interpretive authenticity. Throughout the data analysis, the team will meet to discuss and review emerging themes and search the accounts that provide contesting views of the same phenomena. Close attention will be paid to the complexity and interactions inherent in the focus group data.[43]

Adverse events

For peer review only - http://bmjopen.bmj.com/site/about/guidelines.xhtml

All adverse events and serious adverse events will be recorded on an adverse event log, within study trial management paperwork, case report forms and REDCap. There will be no formal interim analysis of data due to the feasibility nature of the trial. The IDMC will review high level safety data. Adverse events will be monitored at least every month, and as needed on an ad hoc basis, to ensure the continuing safety of the participants. The Scientific Committee will determine the need to terminate the trial. Participants who experience any such event will be directed to the appropriate hospital and all the necessary care will be ensured and followed-up until the participant has resolved or stabilized.

Ethics and dissemination

Ethical approval was obtained from the ethics review committee of the Faculty of Medical Sciences, University of Sri Jayewardenepura (FMS/USJP ERC 64/19) and the University of Leicester, United Kingdom (26770). Further permission for the proposed study will be obtained from the relevant authority at the Central Chest Clinic, Colombo. Sri Lanka. Privacy and the confidentiality of all information and identities of participants will be strictly maintained and will not be disclosed when publishing the results of the study. A copy of the test results obtained will be provided to each participant at the end of the trial for subsequent follow-up and treatment at the clinic. Compensation for travelling will be provided to all the participants. All study documents will be translated to Sinhala and Tamil to ensure clear communication. Participation will be without compulsion and each participant has the right to withdraw at any time, without providing a reason. Consent form and data sheets will be securely stored in a separate locked cupboard. Study computers will be password protected. The participants (adults living with COPD) of the phase 1 study also will be provided the opportunity to participate the PR. All the data will be stored safely up to 6 years and after 6 years consent form and data sheets will be disposed of appropriately. Study team and IDMC only will have access to final trial dataset. Data from the Global RECHARGE Core Dataset will be made available following the completion of this project and we are considering the best tools to use to make this database available to the wider community. Any modifications of the protocol will be updated on trial registry (ISRCTN) and will be informed to the ethics review committee and the participants. Participants will be

For peer review only - http://bmjopen.bmj.com/site/about/guidelines.xhtml

provided the provisions for re-consenting after any change of the approved protocol. It will be made clear in the publication of trial findings.

Author affiliations

[1]Faculty of Health Sciences, KAATSU International University, Sri Lanka
[2]Centre for Exercise and Rehabilitation Science, NIHR Leicester Biomedical Research Centre-Respiratory, University Hospitals of Leicester NHS Trust, UK
[3]Department of Respiratory Sciences, University of Leicester, Leicester, UK,
[4]Sports Medicine Unit, Colombo South Teaching Hospital, Kalubowila, Sri Lanka
[5]Department of Nursing and Midwifery, Faculty of Allied Health Sciences, University of Sri Jayewardenepura, Sri Lanka
[6] National Hospital of Sri Lanka and Central Chest Clinic, Colombo, Sri Lanka
[7]Primary Care Respiratory Group, Sri Lanka
[8]Faculty of Health, University of Plymouth, UK
[9]School of Sport, Exercise and Health Sciences, Loughborough University, UK
[10]Department of Economics, University of Sheffield, Sheffield, UK
[11]Faculty of Medical Sciences, University of Sri Jayewardenepura, Sri Lanka

Author contribution

All authors (Akila R Jayamaha, Chamilya H Perera, Mark W Orme, Amy V Jones, Upendra K D C Wijayasiri, Thamara D Amarasekara, Ravini de S Karunatillake, Amitha C Fernando, Seneviratne A L P De S., Andy Barton, Rupert Jones, Zainab K Yusuf, Ruhme B Miah, Dominic Malcolm, Jesse A Matheson, Robert C Free, Adrian Manise, Michael C Steiner, Savithri W Wimalasekera, Sally J Singh) have substantially contributed to the conception and design of the study. Akila R Jayamaha drafted the manuscript. All authors of the paper have revised the

For peer review only - http://bmjopen.bmj.com/site/about/guidelines.xhtml

content and approved the final version to be published. All authors are accountable for all aspects of the work.

Funding This research was funded by the National Institute for Health Research (NIHR) (17/63/20) using UK aid from the UK Government to support global health research. The views expressed in this publication are those of the author(s) and not necessarily those of the NIHR or the UK Department of Health and Social Care.

Competing interests None declared.

[insert figure caption]

Figure 1: Flow of participant of the three phases of the study

References

1. Singh D, Agusti A, Anzueto A, Barnes PJ, Bourbeau J, Celli BR, et al. Global Strategy for the Diagnosis, Management, and Prevention of Chronic Obstructive Lung Disease: the GOLD science committee report 2019. *Eur Respir J*. 2019;53(5).
2. Alwan A. Global Status Report on Non-Communicable Diseases. WHO, 2010
3. World Health Organization. Global surveillance, prevention and control of Chronic Respiratory Diseases, Switzerland, 2007; 22-23.
4. Amarasiri L, Fernando A, Gunasekara K, Gunasinghe W, Madegedara D, Wickramasinghe R. The prevalence of Chronic Obstructive Pulmonary Disease in Sri Lanka. *Europian Respiratory Journal*, 2017; 2017(50):1212.
5. World Health Organization. WHO strategy for prevention and control of chronic respiratory diseases, Genewa, 2017.
6. Yasaratne D, Dharmage SC. Letter from Sri Lanka. *Respirology*. 2020;25:339-41.
7. Pinnock H, Kendall M, Murray SA, Worth A, Levack P, Porter M, et al. Living and dying with severe chronic obstructive pulmonary disease: Multi-perspective longitudinal qualitative study. *BMJ*. 2011;342:268.

For peer review only - http://bmjopen.bmj.com/site/about/guidelines.xhtml

8. Singh SJ, Halpin DMG, Salvi S, Kirenga BJ, Mortimer K. Comment Exercise and pulmonary rehabilitation for people with chronic lung disease in LMICs : challenges and opportunities. *Lancet Respir* [Internet]. 2019;7(12):1002–4.

9. Nici L, Donner C, Wouters E, Zuwallack R, Ambrosino N, Bourbeau J, et al. American Thoracic Society Documents American Thoracic Society / European Respiratory Society Statement on Pulmonary Rehabilitation. *Am J Respir Crit Care Med*. 2006;173(May):1390-413.

10. Mccarthy B, Casey D, Devane D, Murphy K, Murphy E, Lacasse Y, et al. Pulmonary rehabilitation for chronic obstructive pulmonary disease (Review). *Cochrane Database Syst Rev*. 2015;2015(2).

11. Jones AW, Taylor A, Gowler H, Kelly NO, Ghosh S, Bridle C. Systematic review of interventions to improve patient uptake and completion of pulmonary rehabilitation in COPD. *ERJ Open Res*. 2017;3.

12. Chan AW, Tetzlaff JM, Altman DG, Laupacis A, Gøtzsche PC, Krleža-Jerić K, et al. Spirit 2013 statement: Defining standard protocol items for clinical trials. *Chinese J Evidence-Based Med*. 2013;13(12):1501–7.

13. Bolton CE, Bevan-Smith EF, Blakey JD, et al. British Thoracic Society guideline on pulmonary rehabilitation in adults: accredited by NICE. *Thorax*. 2013;68:ii1-ii30.

14. Ries AL, Bauldoff GS, Casaburi R, Mahler DA, Rochester CL, Herrerias C. Pulmonary Rehabilitation; Joint ACCP/AACVPR Evidence-Based Clinical Practice Guidelines. *Chest*. 2007;131(5):4S-42S.

15. Blackstock FC, Evans RA. Rehabilitation in lung diseases: 'Education' component of pulmonary rehabilitation. *Respirology*. 2019;24(9):863–70

16. Spencer LM, McKeough ZJ. Maintaining the benefits following pulmonary rehabilitation: Achievable or not? *Respirology*. 2019;24(9):909–15.

17. Armstrong M, Vogiatzis I. Personalized exercise training in chronic lung diseases. *Respirology*. 2019;24(9):854–62.

18. Revill SM, Morgan MDL, Singh SJ, Williams J, Hardman AE. The endurance shuttle walk : a new field test for the assessment of endurance capacity in chronic obstructive pulmonary disease. *Thorax*. 1999;54(2):213–22

19. Yasuda H, Yamaya M, Nakayama K, Ebihara S, Sasaki T, Okinaga S, et al. Increased

For peer review only

For peer review only - http://bmjopen.bmj.com/site/about/guidelines.xhtml

Arterial Carboxyhemoglobin Concentrations in Chronic Obstructive Pulmonary Disease. *Am J Respir Crit Care Med*. 2005;171:1246–51.

20. Durnin JVGA, Womersley J. Body fat assessed from total body density and its estimation from skinfold thickness: measurements on 481 men and women aged from 16 to 72 Years. *Br J Nutr*. 1974;32:77–96.

21. Doña E, Olveira C, Palenque FJ, Porras N, Dorado A, Martín-valero R, et al. Body Composition Measurement in Bronchiectasis: Comparison between Bioelectrical Impedance Analysis, Skinfold Thickness Measurement, and Dual-Energy X-ray Absorptiometry before and after Pulmonary Rehabilitation. *J Acad Nutr Diet.* 2018;118(8):1464–73.

22. Lanerolle MD, Lanerolle P, Atukorala S, Silva A De. Enhancing the accuracy of bio impedance analysis in assessing total body water. *Int J Body Compos* Res. 2012;10(1):29–34.

23. Bestall JC, Paul EA, Garrod R, Garnham R, Jones PW, Wedzicha JA. Usefulness of the Medical Research Council (MRC) dyspnoea scale as a measure of disability in patients with chronic obstructive pulmonary disease. *Thorax*. 1999;54:581–6.

24. Herdman M, Gudex C, Lloyd A, Janssen M, Kind P, Parkin D, et al. Development and preliminary testing of the new five-level version of EQ-5D (EQ-5D5L). *Quality of Life Research*. 2011;20(10):1727-736.

25. Van der Molen T, Willemse BW, Schokker S, et al. Development, validity and responsiveness of the Clinical COPD Questionnaire. *Health Qual Life Outcomes.* 2003;1:13.

26. Papaioannou M, Pitsiou G, Manika K, Kontou P, Zarogoulidis P, Sichletidis L, Kioumis IP. COPD assessment test: A simple tool to evaluate disease severity and response to treatment. *J of Chronic Obstructive Pulmonary Disease*. 2014;11:489- 95.

27. Reilly MC, Zbrozek IAS, Dukes EM. The Validity and Reproducibility of a Work Productivity and Activity Impairment Instrument. *Pharmacoeconomics*. 1993;4(5):353–65.

28. Zigmond AS, Snaith RP. The hospital anxiety and depression scale. *Acta Psychiatrica Scandinavica.* 1983;67(6):361-70.

29. Buysse DJ, Reynolds CF, Monk TH, et al. The Pittsburgh Sleep Quality Index: a new instrument for psychiatric practice and research. Psychiatry Res. 1989;28:193–213

30. Weisman IM, Marciniuk D, Martinez FJ, Sciurba F, Sue D, Myers J, et al. American Thoracic Society / American College of Chest Physicians ATS / ACCP Statement on Cardiopulmonary Exercise Testing. *Am J Respir Crit Care Med*. 2003;167:211–77.

For peer review only - http://bmjopen.bmj.com/site/about/guidelines.xhtml

31. Singh SJ, Morgan MD, Scott S, Walters D, Hardman AE. Development of a shuttle walking test of disability in patients with chronic airways obstruction. *Thorax*. 1992;47(12):1019-24.

32. Mahler DA, Horowitz MB. Perception of breathlessness during exercise in patients with respiratory disease. *Med Sci Sports Exerc*. 1994;26:1078–1081.

33. Borg GA. Psychophysical bases of perceived exertion. *Med Sci Sports Exerc*. 1982;14:377–381

34. Jones SE, Kon SSC, Canavan JL, Patel MS, Clark AL, Nolan CM, et al. The five-repetition sit-to-stand test as a functional outcome measure in COPD. *Thorax*. 2013;68:1015–20.

35. Booth M: Assessment of physical activity: An International perspective. *Research Quarterly for Exercise and Sport*. 2000, 71 (2): 114-120.

36. Bassett DR, John D. Use of pedometers and accelerometers in clinical populations: validity and reliability issues. *Phys Ther Rev*. 2010;15:135–42.

37. Choi L, Liu Z, Matthews CE, Buchowski MS. Validation of accelerometer wear and nonwear time classification algorithm. *Med Sci Sports Exerc*. 2011;43:357–64.

38. Demeyer H, Burtin C, Van Remoortel H, Hornikx M, Langer D, Decramer M, et al. Standardizing the analysis of physical activity in patients with COPD following a pulmonary rehabilitation program. *Chest* 2014 Aug;146(2):318-327

39. Troiano RP, Berrigan D, Dodd KW, Mâsse LC, Tilert T, McDowell M. Physical activity in the United States measured by accelerometer. *Med Sci Sports Exerc* 2008 Jan;40(1):181-188.

40. Lucas AO, Gilles HM. Approaches to economic evaluation. *Short Textbook of Public Health Medicine for the Tropics.* 2002. CRC Press.

41. Harris PA, Taylor R, Minor BL, Elliott V, Fernandez M, Neal LO, et al. The REDCap consortium: Building an international community of software platform partners. *J Biomed Inform*. 2019;95.

42. Braun V, Clarke V. Using thematic analysis in psychology.*Qual Res Psychol*. 2006;3:77–101

43. Maguire M, Delahunt B. Doing a Thematic Analysis : A Practical, Step-by-Step Guide for Learning and Teaching Scholars. *AISHE-J*. 2017;8(3):3351–14.

For peer review only - http://bmjopen.bmj.com/site/about/guidelines.xhtml

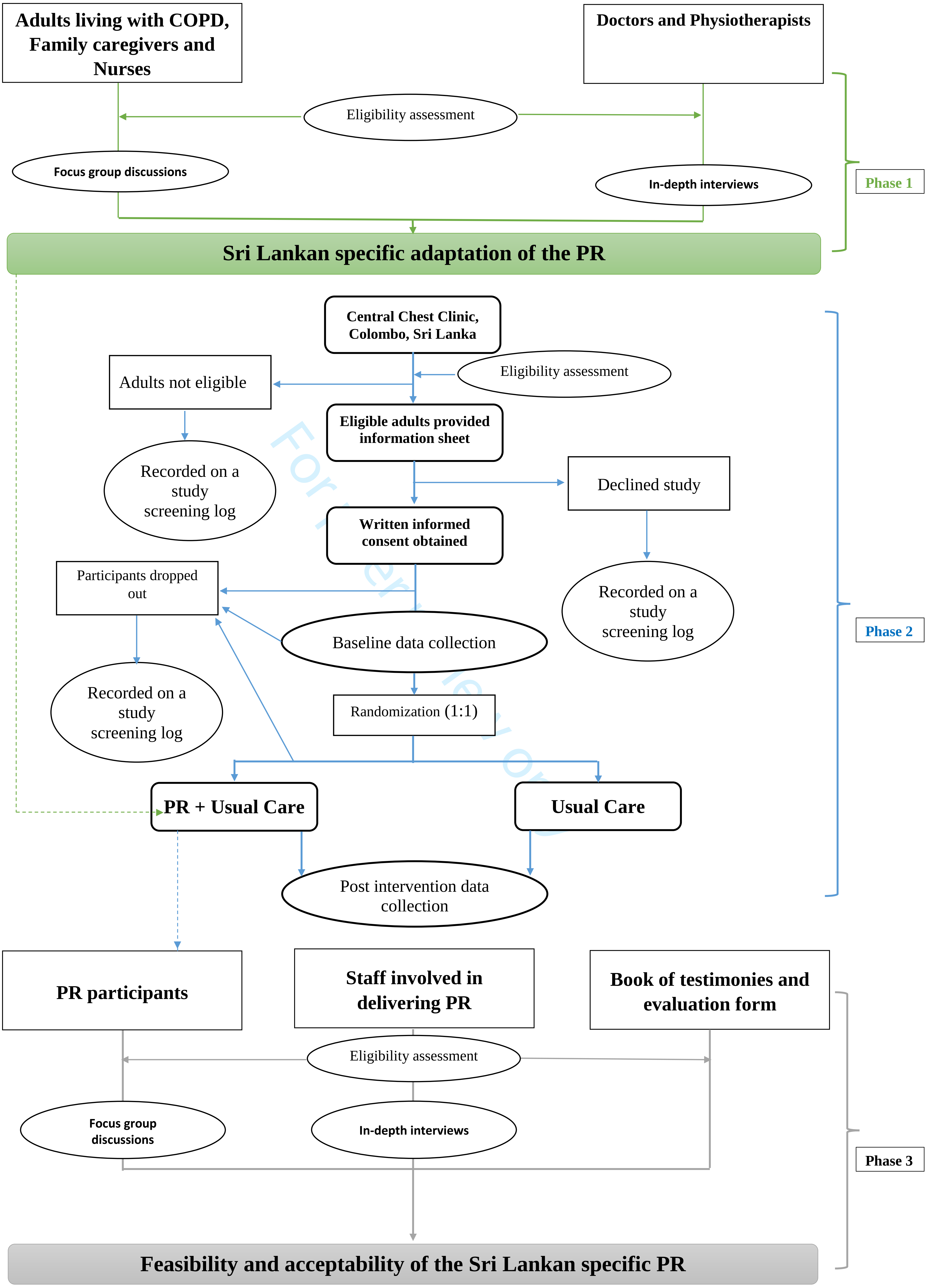
Adults living with COPD, Family caregivers and Nurses
Doctors and Physiotherapists
Eligibility assessment
Focus group discussions
In-depth interviews
Phase 1
Sri Lankan specific adaptation of the PR
Central Chest Clinic, Colombo, Sri Lanka
Adults not eligible
Eligibility assessment
Eligible adults provided information sheet
Recorded on a study screening log
Declined study
Written informed consent obtained
Participants dropped out
Recorded on a study screening log
Baseline data collection
Recorded on a study screening log
Randomization (1:1)
PR + Usual Care
Usual Care
Phase 2
Post intervention data collection
PR participants
Staff involved in delivering PR
Book of testimonies and evaluation form
Eligibility assessment
Focus group discussions
In-depth interviews
Phase 3
Feasibility and acceptability of the Sri Lankan specific PR

Appendices

Information sheet

Cultural adaptation of Pulmonary Rehabilitation and subsequent testing in a Randomised Controlled Feasibility Trial for Adults with Chronic Obstructive Pulmonary Disease in Sri Lanka

I am Prof. S, W. Wimalasekera, of Department of Physiology, Faculty of Medical Sciences, University of Sri Jayewardenepura. I and my team of health care personnel are, conducting a research on "Testing the Feasibility and Acceptability of a Culturally Appropriate Hospital-Based Pulmonary Rehabilitation (PR) Programme for Adults with Chronic Obstructive Pulmonary Disease in Sri Lanka". I invite you to participate in the study. Please take time and read the following information carefully before giving your consent. You are free to ask questions to clarify facts which are not clear or ask for more information if you need.

Purpose of the study

The purpose of this study is to devise an appropriate PR programme and then determine the feasibility and acceptability of this programme for adults living with COPD in Sri Lanka and assess the potential for a future trial of its effectiveness.

When you participate you will be required to do the following

You will be requested to participate several components of the study.

You will participate in a group discussion conducted in a quiet room in the clinic (conference hall) with very minimal disturbances. At the beginning of the discussion you will answer some questions regards your personal information, (age, gender, history of the disease condition). Then the interviewer will ask some questions regarding your thoughts and ideas about pulmonary rehabilitation. You and your group members can freely comment on the questions or can add your ideas to the discussion. The interview takes around forty-five minutes of time.

You will also participate in a feasibility trial which you will be allocated to one of the two groups. One group will receive usual care and PR and the other group will receive only usual care which consists of pharmacological treatment and brief information about disease condition, medication and inhaler techniques. PR is typically a 6-week rolling programme and consisting of 12 sessions in total, delivered twice a week. Sessions usually last approximately two hours, with one hour for exercise training and one hour for education. Some routine and specific test and measurements will be taken before and after the intervention will describe any changes in the health of the adults living with COPD.

If you are allocated to the group who receive the PR, you will be asked to share your experiences regarding the PR and subjected modification and opinions on pulmonary rehabilitation.

In order to maintain accuracy, and to keep records, your discussion will be recorded by the research team members with your permission. Your participation in the project will remain confidential and the provision of your name and address is necessary for us to maintain records. Any personal information gathered during the study will not be disclosed to a third party. Your responses to the questions and will

For peer review only

only be used for the purpose of this project. You can be assured that you will remain anonymous if you take part in the project. Your participation in this project is entirely voluntary. You are not obliged to take part. If you do not wish to take part, you can withdraw without giving a reason and your medical care will not be affected, further you will not be contacted again. Similarly, if you do agree to participate, you are free to withdraw at any time during the project.

What are the possible benefits of taking part?

By participating in the study, you will contribute to develop a PR programme which helps to improve the quality of life of the adults living with COPD. As well as you will contribute to determine the feasibility and acceptability of this programme within the health care system in Sri Lanka.

What are the possible disadvantages and risks of taking part?

You will be undertaking some physical tests as part of the research. Therefore, there may be a very small risk of falls, breathlessness, changes in blood pressure and changes in heart rate. However, these risks are very rare, and trained staff and emergency equipment will be available to deal with any serious events. Participants who experience any such event will be directed to the appropriate hospital and all the necessary care will be ensured and followed-up until the participant has resolved or stabilized.

What will happen if you don't carry on with the study?

If you withdraw from the study all the information and data collected from you, to date, will be destroyed and your name would be removed from all the research study files. There will not be any loss or impact on your routine medical care which you are entitled to.

What will happen to the results of the research study?

The results of all the participants of the study will be used for scientific research. All data will be available only to the researchers and information provided will be strictly confidential. Your participation in the study is highly appreciated. Privacy and confidentiality of data gathered is completely secure and will be used only for research purposes. If you have any questions or any concerns with regards to the study and need further information you can call the telephone number listed below. Further you can send any complaint to the above postal address or email address.

Yours faithfully

Prof. S.W. Wimalasekera

Department of Physiology, Faculty of Medical Sciences, University of Sri Jayewardenepura

T.P No. 0777487203 e-mail : savithriww@yahoo.com

Ethics Review Committee – University of Sri Jayewardenepura - +94 11 2758000 Ext 4075e

For peer review only

Consent Form: **Randomised Controlled Feasibility Trial**

Cultural adaptation of Pulmonary Rehabilitation and subsequent testing in a Randomised Controlled Feasibility Trial for Adults with Chronic Obstructive Pulmonary Disease in Sri Lanka

1.Have you read the information sheet? (please keep a copy for yourself) YES/NO

2.Have you had an opportunity to discuss this and ask any questions? YES/NO

3. Have you had satisfactory answers to all your questions? YES/NO

4. Have you received enough information about the study? YES/NO

5. Who explained the study to you---

6. Do you understand that you are free to withdraw from the study at any time,

without having to give a reason YES/NO

7. All personal details will be treated as STRICTLY CONFIDENTIAL.

Do you give your permission for these individuals to have access to the records? YES/NO

8. Have you had sufficient time to come to your decision? YES/NO

9. Do you agree to take part in this study? YES/NO

Participant’s signature --------------------------------------- Date------------------------

By the Witness

I have been present while the procedure was explained to the participant and I have witness his/ her willingness to take part in the study.

Signature of witness-- Date-------------------------------

(The witness should be a person not connected to the study)

Name --

Contact details--

For peer review only
